# Evaluating the feasibility and preliminary impact of the Social, Emotional, and Ethical (SEE) Learning program: A compassion-based social and emotional learning program for elementary school children

**DOI:** 10.1371/journal.pone.0328519

**Published:** 2025-08-13

**Authors:** Tyralynn Frazier, Robert W. Roeser, Kimberly A. Schonert-Reichl, Lobsang Tenzin Negi

**Affiliations:** 1 The Center for Contemplative Science and Compassion-Based Ethics, Emory University, Atlanta, Georgia, United States of America; 2 Bennett Pierce Professor of Caring and Compassion, Professor of Human Development and Family Studies, The Pennsylvania State University, State College, Pennsylvania, United States of America; 3 NoVo Foundation Endowed Chair in Social and Emotional Learning, Professor in the Department of Psychology, University of Illinois Chicago, Chicago, Illinois United States of America; University of Sharjah College of Health Sciences, UNITED ARAB EMIRATES

## Abstract

**Objectives:**

This study aimed to assess the feasibility and preliminary outcomes of the SEE Learning® (Social, Emotional, and Ethical Learning) program among elementary school-aged children.

**Methodology:**

A quasi-experimental design was employed, with 685 4^th^- and 5^th^-grade students across 33 classrooms (344 students received the 12-week SEE Learning program; 341 students were wait-list controls). Assessments of compassion for self and others, social and emotional competencies, and the degree to which students perceived their classrooms as supportive were collected before and after program implementation. Measures of dosage, fidelity, and acceptability were assessed via teachers’ weekly lesson diaries.

**Results:**

Teachers reported the program was feasible to implement. They demonstrated high lesson completion and fidelity rates with minimal preparation time and strong adherence to the lesson structure. Most program activities fit within a 50-minute timeframe or less, reflecting the program’s suitability for elementary school settings. Student reports showed preliminary impacts of the program. Those who received SEE Learning reported significant improvements in self-compassion, perspective-taking, empathic concern (e.g., compassion for others), intrinsic prosocial motivation, and academic goal setting compared to students in the wait-list control group.

**Significance:**

This study is among the first to demonstrate the feasibility and preliminary student impacts of the compassion-focused SEE Learning program in an elementary school setting. Future investigations might explore the implementation and effects of the SEE Learning program using randomized-controlled experimental designs and longer-term follow-ups. In addition, studies evaluating program implementation and impacts in diverse cultural-contextual settings, and among students of different ages, are needed. In sum, the SEE Learning program shows evidence of promise for impacting elementary school students’ prosocial skills and competencies.

## Introduction

Social and emotional learning (SEL) plays a crucial role in education, encompassing knowledge, attitudes, and skills necessary to recognize and manage emotions, develop empathy, establish positive relationships, and make responsible decisions [1–5]. School-based SEL programs have gained prominence globally, demonstrating positive effects on students’ social and emotional competencies, mental health, and academic achievement [5–8]. Research findings have consistently shown the positive benefits of SEL programs in increasing students’ social and emotional competencies, health, and well-being, as well as decreasing anxiety, depressive symptoms, and stress [9,10]. Moreover, SEL programs have demonstrated significant impacts on students’ academic achievement. Longitudinal studies also demonstrate that SEL programs provide children with essential life skills that extend beyond their schooling and are sustained into adulthood [11,12]. By nurturing young learners’ emotional and social development, SEL programs lay the foundation for more positive outcomes in students’ academic, social, and emotional development.

### Contemplative approaches in education

More recently, educational programming and research have extended SEL work to include contemplative approaches, such as direct attention training and prosocial emotions such as compassion [13–17]. Most of this research has centered on mindfulness-based interventions (MBIs) [18–22], examining how specific practices, such as mindful movement and body scanning to enhance self-awareness, and loving-kindness practices like gratitude journaling and encouraging acts of kindness, support the development of students’ attentional and emotional skills. These skills are closely linked to self-regulation, prosocial behavior, and positive outcomes, including academic achievement and healthy social relationships [19–21,23,24].

The theory of change for this work is that developmental windows of opportunity exist in the early decades of life when the plasticity of neural and mental systems is particularly high [25]. Mental training and enrichment that targets attentional, social, and emotional skills through practices like reflection, focused attention practices, and engaging in service activities during such windows of opportunity are hypothesized to be particularly impactful for cultivating skills and outcomes during early to late childhood [13,24,26,27]. Several studies in elementary school students show promising program outcomes, including improvements in attention, executive functioning, working memory, and emotional regulation [18,19,28,29].

The elementary school period, particularly late elementary, is a crucial developmental stage during which children develop their capacities for self-control, perspective-taking, empathy, and prosocial behavior [30,31]. Early research suggests that this juncture, around ages 5–10, presents an optimal opportunity for implementing compassion-based programs in schools [32–35]. By introducing such programs during these years, educators and researchers can tap into this critical period of neurodevelopment to foster greater social and emotional skills in young individuals, potentially yielding lasting positive effects on their overall well-being and social interactions [33–39]. The work in contemplative education fills a gap in the SEL programming by focusing on the direct cultivation of attention, awareness, and compassion.

The specific programs and practices used in MBI studies vary, but they often include structured mindfulness curricula such as *MindUP* [21], the *Kindness Curriculum* [28], and the *Attention Academy Program* [20]. These programs typically incorporate age-appropriate breathing exercises, body scans, mindful movement (such as yoga), and guided meditations. Some MBIs also incorporate elements of positive psychology and SEL, including activities like gratitude journaling, loving-kindness practices, and reflective discussions. For example, the *MindUP* curriculum integrates neuroscience and mindfulness to help students understand how their brains respond to stress and how to regulate their emotions [21]. Similarly, the Attention Academy emphasizes building sustained attention through daily mindfulness exercises. These practices are typically delivered in brief, consistent sessions integrated into the school day, making them accessible and developmentally appropriate for elementary school children [20]. Moreover, emerging research suggests that integrating mindfulness directly into the core curriculum, rather than treating it as a separate activity, may be more effective. Studies have shown that curriculum-based approaches can enhance students’ self-awareness, present-moment focus, and engagement through experiential learning [40].

### SEE learning framework

**Fig 1:**
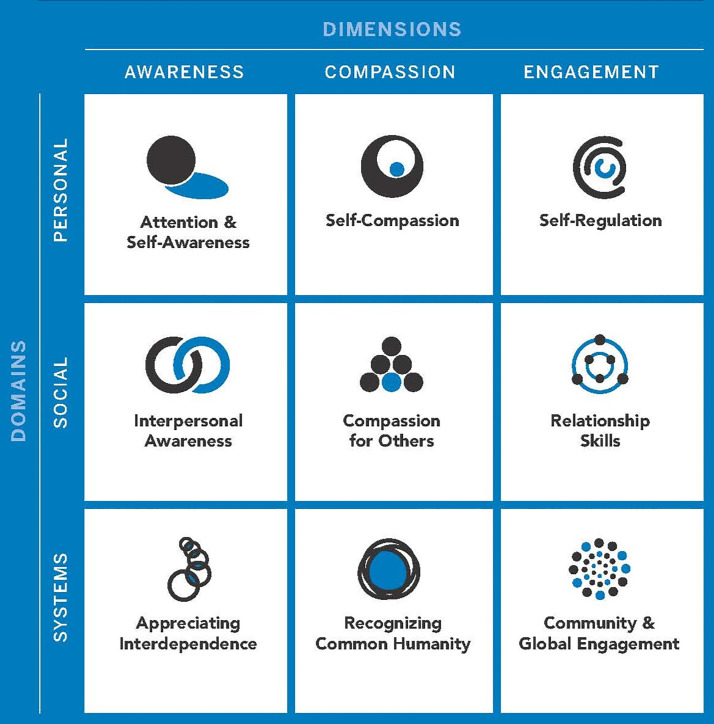
The SEE learning framework. The SEE Learning framework brings together compassion-based ethics, SEL, and key competencies for ethical development. It nurtures students’ growth in areas of attention, compassion, resilience, and systems thinking, fostering overall well-being and ethical understanding. This framework is the foundation from which the SEE Learning curriculum was developed.

As stated above, current contemplative practices emphasize mindfulness. Compassion-based approaches, such as those introduced in SEE Learning, offer additional, developmentally appropriate support by explicitly cultivating ethical awareness, relational empathy, and systems thinking. These practices go beyond self-regulation to foster a deeper understanding of interdependence and common humanity, aligning more closely with the holistic developmental needs of young learners. The program, called Social, Emotional, and Ethical Learning or SEE Learning, is a new SEL program comprised of a compassion-based educational framework. It was developed by Emory University’s Center for Contemplative Science and Compassion-Based Ethics. The program was designed to foster the holistic development of students by cultivating qualities of attentional awareness, purposeful engagement, and compassion cultivation across intrapersonal, interpersonal, and systems perspectives of experience [2] (Fig 1). Compassion can be defined as “a sensitivity to suffering in self and others with a commitment to try to alleviate and prevent it” (p. 11) [41].

**Fig 2:**
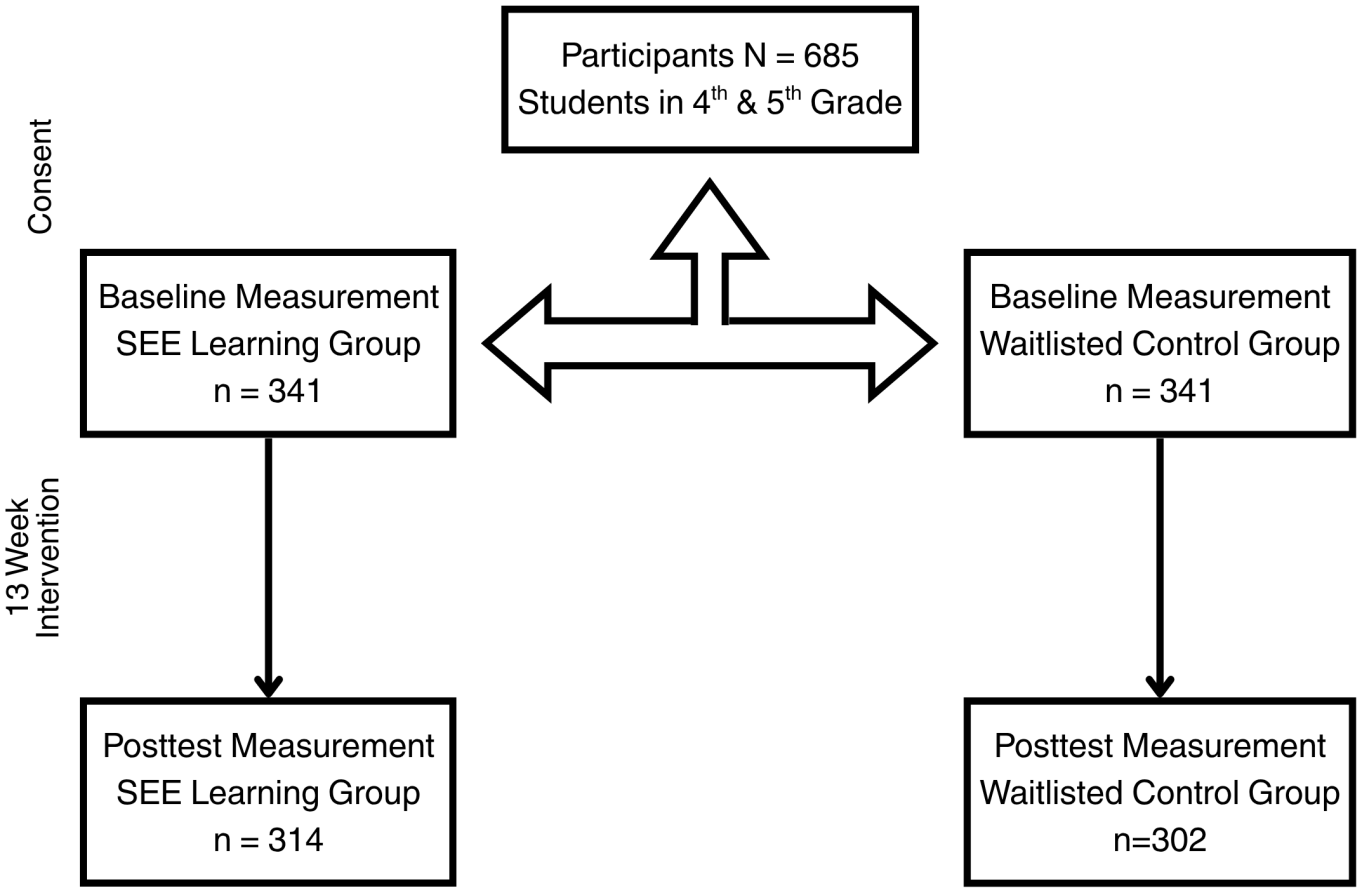
Participant flowchart. This diagram illustrates participant retention across pre-test or baseline and post-test study timepoints between the SEE Learning Group and the Waitlisted Control Group.

The goal of the SEE Learning framework is to cultivate dispositions and skills necessary for the development of compassionate individuals endowed with ethical discernment and compassionate engagement with the world, others, and self (Fig 1). This is hypothesized to be achieved through the cultivation of three different dimensions: awareness, compassion, and engagement, and in relation to three levels of analysis or domains: the personal, social, and systems-level. Through practices such as focused attention or open awareness meditation, the practitioner develops awareness, including awareness of self, others, and the social and natural worlds in which one is embedded [41,42]. Through compassion practices, one develops empathy and compassion for oneself and others, as well as the recognition of our common humanity in which we value all human beings and all sentient beings [43,44]. Through engagement with the world and other people, one learns self-regulation and restraint, kindness and other-orientation, and an appreciation of contributing to the welfare of wider and wider communities and social systems [43,45–47]. The three domains are often referred to as the head, the heart, and the hand. Across these three dimensions and domains, we see the SEE Learning Framework’s nine key conceptual themes and components or competencies (Fig 1).

Commencing with the cultivation of awareness and empathy towards the suffering of others, students explore the broader concepts of compassion and kindness. Subsequently, they delve into emotional awareness and resonance as they discover compassion as an intrinsic quality within themselves. This learning phase is intertwined with the development of tolerance for discomfort, a crucial facet of compassion development. As the program unfolds, students are empowered to harness internal motivation to alleviate suffering and make informed choices for compassionate engagement.

### The current study

The primary purpose of this study was to investigate the implementation and preliminary impact of the SEE Learning program on 4^th^ and 5^th^-grade elementary children aged 9–11. The specific study aims were twofold and included the following:

1] To assess the implementation (feasibility, dosage, acceptability) of the SEE Learning program in classrooms with upper elementary school students; and
2] To examine the preliminary impacts of the SEE Learning program on student outcomes regarding dimensions associated with the SEE Learning Framework (Fig 1).

## Materials and methods

### Ethical statement

Emory University’s Ethical Review Board reviewed and approved this study. The approval number is STUDY00002823. Informed consent was obtained from students and parents. For the parents, the intervention and research study were described to parents/guardians via email communication, school newsletters, and an open question-and-answer session. All students in the participating classrooms were invited to participate in the assent and parental consent process. Parental consent occurred via passive consent, with parents choosing to opt their children out of study participation. Student assent occurred via active informed consent in the classroom prior to participation in the assessment process. Students were initially introduced to the study during an information session before the study period began. On the day of assessment, all students were given detailed instructions on study participation. The consent form was read out loud, and the students were asked to check a box on a written form indicating their consent decision. This protocol was based on standard practices that have been previously used across education-based studies. Students who chose not to consent were provided an alternative activity during the assessment process. The SEE Learning program was provided to all students in the participating classrooms, regardless of consent or assent. Consent and assent were obtained only for students to complete the study measures.

### Research design

The current study was conducted via a quasi-experimental research design, with pre-tests and post-tests. After pre-test assessments, the SEE Learning program was implemented over 13 weeks to 4^th^ and 5th-grade students (*N* = 685) in 17 classrooms across two schools, while students in 16 classrooms in two other schools served as waitlist controls. The schools in this study were matched by race and socioeconomic diversity. The district administrators were contacted and introduced to the SEE Learning program via a short introductory session. After the introduction to the program, the district administrators shared the study flyer with school principals. Recruitment began May 1^st^, 2022, and continued until October 1^st^, 2023.

#### Recruitment of participants.

The participating schools were in the Southeastern USA. The demographic profiles of the public and private schools in this study demonstrate similarities in minority student enrollment, gender distribution, and student-teacher ratios across the participating classrooms. After approval by the relevant University Institutional Review Board, school principals who received flyers and agreed to meet participated in a one-on-one meeting with research study team personnel. Once the principals agreed to participate in the research, they provided the study team with a letter of support and gave the study team permission to reach out to teachers. Participation in the program was voluntary across all schools. All classroom teachers were involved in implementing the program, regardless of whether they participated in the study. Study participation specifically involved completing research questionnaires. The control classrooms were able to implement the program after the study was completed. After the administrator meeting and approval, teachers received the recruitment flyer describing the research study. Teachers who expressed interest in participating met with a member of the research study team for a one-on-one meeting. During this period, study team members guided teachers through the consent and assent process to ensure they fully understood what participation in the assessments entailed. Volunteering specifically referred to allowing access to their students for consent to complete the study-related measurements, and for teachers to participate in study-related implementation tracking. Importantly, all schools, whether in the implementation or waitlisted control groups, had already planned to implement SEE Learning, independent of their participation in the study.

Of the 33 teachers, 17 were selected to receive the SEE Learning program training, and 16 were selected to serve as wait-listed controls and receive the program training in the subsequent school year. The selection of schools participating in this study involved a convenience sample of schools interested in the SEE Learning program. Teachers were recruited on a first-come, first-served basis, with the first schools approached being recruited to implement SEE Learning in the classrooms. We then approached teachers for recruitment to serve as wait-listed controls. If the teacher agreed to have their classroom participate in either group, they participated in an additional meeting with a member of the study team to discuss logistics and planning for data collection.

After the one-on-one meeting where teachers consented to the study, the project was described to parents/guardians via email communication, school newsletters, and an open question-and-answer session that was conducted virtually with research study team members. Information about the research study was also shared with students through in-person classroom visits by members of the research team. All 4^th^ and 5th-grade students in participating classrooms were invited to participate in the student assent and parental/guardian consent process. Parental/guardian consent occurred via a passive consent procedure in which parents/guardians were notified of the research study and had the opportunity to proactively decline to allow their children’s participation.

Overall, there was a 99% participation rate regarding parent/guardian passive consent across all classrooms. On the day of pre-test data collection, students were guided through a process of student assent by members of the research study team. After a review of the study’s purpose and participation guidelines, all students completed a form that indicated whether they chose to participate in the study. Student assent ranged from 85%−99% across all classrooms at pre-test. At post-test, there was a 9% participant attrition in the intervention group and an 11% participant attrition in the control group (Fig 2). The post-test for both the SEE Learning program and control classrooms was conducted within three weeks after the implementation period, at the end of the school year. Assessments took place after the final tests and exam periods had concluded. All participating teachers received a small honorarium of $50.

#### Sample size.

We used G*Power to compute the sample size, considering a medium effect size (0.25), α = 0.05, and β = 0.2 [48]. We were also guided by the National Center for Education Evaluation [49]. The standard target for an education-based intervention study is a sample size of 300 students (150 in each group) to ensure statistical significance at a 0.05 alpha level, assuming only a 1 in 20 chance of a null effect. To meet the requirements of our main study while considering potential attrition, we aimed for an equivalent sample of 350 students in each group. However, to better reflect the nuances of SEL research, we revised our sample size calculations based on recent meta-analytic findings [9]. We adopted a conservative effect size of Cohen’s d = 0.15, informed by a comprehensive meta-analysis of SEL programs. This adjustment, coupled with an alpha level of α = 0.05 and a power of 1 – β = 0.775, led us to a target sample size of 400 students (200 per group). This increase accounts for potential attrition and bolsters our study’s capacity to detect statistically significant effects, thereby enhancing the validity of our findings.

#### Participants.

The final sample included 685 4^th^ and 5th-grade children and their classroom teachers in two public schools and one private school. A total of 344 4^th^ and 5th-grade students were in the SEE Learning implementation group, and 341 4th and 5th-grade students served as waitlist controls (Table 1). The intervention group included a higher percentage of 4th graders (60.4%) compared to the control group, which had a larger representation of 5th graders (52.6%). This difference in grade distribution was statistically significant, as indicated by a chi-squared test (Χ2 = 11.6, *p* < 0.001). Gender distribution was similar in both groups. Similarly, there were marginal and non-significant differences in English proficiency between the groups, with high-proficiency students being predominant in both the intervention group (98.2%) and the control group (99%). When considering race and ethnicity, differences emerged. When asked about race, students were able to self-identify, and they were not limited to one choice. The intervention group had a higher percentage of self-identifying Asian students (9.9%) compared to the control group (4.1%). The control group, on the other hand, had a higher percentage of self-identifying Black students (21.4%) compared to the intervention group (15.5%). White students made up a substantial proportion in both groups without significant differences. In terms of family composition, most participants in both groups had two or more parents, and this did not differ significantly. Although differences by race were detected, controlling for race did not affect the outcomes of the analysis; thus, only grade level was controlled for in subsequent analyses (Table 1).

**Table 1:** Descriptive statistics for demographic variables with t-test for mean comparisons and Chi-Squared test for group difference of percentages.

Variables	Intervention (*n* = 344)	Control (*n *= 341)	Total (N = 685)	Difference
				**X2 (p-value)**
**Age (mean, SD)**				
	10 (1)	10 (1)	10 (1)	0.99
**Grade (%)**				
4^th^	60.4	47.4	53.8	11.6 (0.00)
5^th^	39.6	52.6	46.2	11.6 (0.00)
**Gender (%)**				
Girls	52.5	53.1	53.0	0.03 (0.88)
Boys	44.5	44.1	44.1	0.03 (0.87)
Nonbinary	3.0	2.8	2.9	0.00 (0.98)
**English Proficiency (%)**				
Low	1.8	1	1.5	0.99 (0.32)
High	98.2	99	98.5	0.98 (0.32)
**Race (%)**				
Asian	9.9	4.1	7.0	0.00 (8.77)
Black	15.5	21.4	18.4	0.04 (4.11)
Hispanic	2.91	2.93	2.92	0.98 (0.00)
White	41.9	42.8	42.3	0.80(0.01)
Other*	23.9	17.3	20.6	0.03(4.47)
**Family Composition (%)**				
Single Parent	14.2	16.4	15.3	0.63(0.43)
Two or More Parents	85.8	83.6	85.7	4.63(0.43)

### Teacher training in the SEE learning program

Prior to the pre-test data collection, school principals facilitated the release of teachers who were assigned to the SEE Learning intervention to attend a three-day training in the SEE Learning program implementation. The training was facilitated by a certified SEE Learning facilitator. This was a three-day training that lasted for four hours each day. The first day consisted of an overview of the program and the conceptual theories and framework guiding the program. This was also a time when the teachers were introduced to practices that they would use for themselves and their own well-being. The following two days included experiential training, where teachers practiced the learning experiences and experienced fishbowl exercises for feedback. During this exercise, teachers had the opportunity to role-play lessons. The final training session was used as planning time for mapping out their classroom implementation of the SEE Learning program. Facilitation quality, participant engagement, and attendance were tracked. Participants’ reports regarding the quality of the training facilitation were very high, with mean levels of engagement and quality reported as high to very high among more than 95% of the teachers. All teachers who were in the intervention group attended 90% or more of the training.

### The classroom implementation

The SEE Learning program was delivered weekly by 4th and 5th-grade classroom teachers over the course of 13 weeks during the spring semester of the academic year. This occurred during the COVID pandemic when children had returned to in-person instruction. The scope and sequence of the learning experiences delivered are listed in Table 2. Teachers delivered the learning experiences to their regular classroom students during a time determined by them each week. Lessons typically lasted between 30 and 55 minutes. This cadence of delivery was consistent over the intervention period. Students in the waitlist control classrooms were exposed to business-as-usual SEL programs following the guidelines of their district or school initiatives. The business-as-usual SEL exposure was comparable across study sites.

**Table 2:** Intervention scope & Sequence of SEE learning curriculum delivered.

	Week 1	Week 2	Week 3	Week 4	Week 5	Week 6	Week 7	Week 8	Week 9	Week 10	Week 11	Week 12	Week 13
**Lesson Title**	Exploring Compassion	Exploring Kindness	Class Agreements	Practicing Kindness & Compassion	Recognizing Compassion & Exploring Interdependence	Exploring Sensations	Resourcing	Creating a Resource Kit	Grounding	The Resilience Zone	Exploring the Resilience Zone through Scenarios	How Compassion & Safety Affect the Body	Teachers used this week to complete any LEs or revisit concepts with students.
**Lesson Objective**	Explore the relationship between happiness, kindness, and compassion	Explore how the universal wish for happiness underlies human action, motivation, and emotion	Identify agreements to create a compassionate classroom	Develop ways of exhibiting compassion in the classroom	Recognize how things that meet our needs come from the actions of countless others	Develop vocabulary describing body sensations and how to identify them	Track sensations in the body and identify a personal resource that calms the body	Create a resource kit they can use to calm themselves when stressed	Gain proficiency in the practice of grounding through different methods	Understand the zones of well-being and recognize which zones they are in at any given moment	Learn to track which zone they are in by tracking body sensations. Use resourcing and grounding to shift.	Recognize how class agreements, kindness, and safety can affect the nervous system, and this can affect physical health	Teacher-Choice

### Measures

#### Procedure.

Students completed self-report measures before their teachers underwent training. Throughout the implementation phase, teachers monitored program delivery by providing weekly feedback on its extent and quality. The final assessment took place at the conclusion of the spring semester, aligning with the end of the academic year and the administration of end-of-year exams, for both intervention and wait-list control groups. All assessments were conducted by research team members.

#### Implementation dosage, fidelity, and acceptability.

Given the importance of monitoring dosage and fidelity in SEL program implementation [49–52], teachers implementing the SEE Learning program were asked to complete weekly implementation diaries [44]. Each week, implementation dosage and fidelity were tracked via implementation feedback diaries that teachers completed after the delivery of each lesson. The diaries asked teachers to report on lesson completion and fidelity, preparation and delivery time, teacher comfort, and student engagement. For example, preparation time gauged the average duration teachers dedicated to lesson planning, ensuring it was manageable and consistent. Lesson fidelity measured the adherence to the lesson plans, with higher percentages indicating fewer deviations from the intended curriculum. Acceptability of the SEE Learning program was assessed via teachers’ comfort level of teachers delivering the lessons. Teachers also rated the quality of student engagement during each lesson on a 5-point scale ranging from 1 (“*not engaged*”) to 5 (“*very engaged*”). Lastly, lesson duration provided information on the time spent on each lesson, highlighting the efficiency of lesson delivery within the classroom setting (S1 Table).

#### Student outcome measures.

The following are the measures administered to all student participants (S2 Table). Students completed eight questionnaires, comprising a total of 114 items. The entire assessment took approximately 35 minutes to complete.

#### Interpersonal reactivity index – perspective-taking.

Students’ perspective-taking was assessed via the Interpersonal Reactivity Index [52] (IRI), which has been modified for children [53]*.* The IRI is a self-report measure consisting of 28 items with four subscales (7 items per subscale): perspective-taking, empathic concern, personal distress, and fantasy. For the purposes of the present study, only the perspective-taking and empathic concern subscales were used. Examples of questions from the perspective-taking subscale include “It’s easy for me to understand why other people do the things they do” and “Even when I’m mad at someone, I try to understand how they feel.” Students rated each item on a 5-point rating scale, ranging from 1 = *Not at all like me* to 5 = *Always like me*. Scores were computed by averaging item scores within subscales so that higher scores signified greater perspective-taking. Supportive evidence for the construct validity of the perspective-taking subscale of the IRI has been obtained in previous research [53], including significant correlations with related constructs in expected directions. For the present study, the perspective-taking subscale demonstrated adequate internal consistency both at the pre-test and post-test. Specifically, Cronbach’s alphas were 0.76 at the pre-test and 0.76 at the post-test.

#### Interpersonal reactivity index – Empathic concern.

The Empathic Concern subscale of the IRI [53] modified for children [54] was used to assess students’ empathy. The Empathic Concern subscale includes seven items that assess the affective component of empathy, specifically the feelings of warmth, compassion, and concern for others. This subscale evaluates the emotional response of individuals to the experiences of others. Example items include “I often feel sorry for people who don’t have the things I have” and “I often feel sorry for other children who are sad or in trouble,” which measure the respondent’s feelings of sympathy and concern. Students rated each item on a 5-point rating scale, ranging from 1 = *Not at all like me* to 5 = *Always like me*. Scores were computed by averaging item scores so that higher scores signified greater empathic concern. As with the perspective-taking subscale of the IRI, there is empirical evidence supporting the construct validity of the empathic concern subscale [55], including significant correlations with empathy-related constructs in predicted directions [55]. For the present study, the empathic concern subscale demonstrated adequate internal consistency both at the pre-test and post-test. Specifically, Cronbach’s alphas were 0.81 at the pre-test and 0.83 at the post-test.

#### Emotional Expression Scale for Children (EESC).

We utilized the Emotional Expression Scale for Children [54] (EESC) to assess students’ ability to tolerate uncomfortable feelings. The EESC serves as a valuable measure of emotional tolerance. The scale used in this study consists of 8 items and utilizes a 5-point Likert scale, ranging from 1 = *Not at all true* to *Extremely true*. Example questions include “I have feelings that I can’t figure out” and **“**I often do not know how I am feeling.” Scores are averaged, with higher scores indicating poorer emotional awareness and greater reluctance to express emotions. In the present study, the scale’s internal consistency is reflected in Cronbach’s alphas of 0.78 at the pre-test and 0.83 at the post-test.

#### The intrinsic prosocial motivation scale.

The Intrinsic Prosocial Motivation scale [56] is a self-report measure consisting of three items. The scale assesses individuals’ motivation to engage in prosocial behaviors. Intrinsic motivation reflects an internal desire to help others based on students’ inclination to act in prosocial ways because of personal feelings of empathy, concern for the others, or commitment to interpersonal growth. An example of intrinsic motivation is “I help because I think it is good to help,” and an example for extrinsic motivation is “I help because I want to get a reward or praise from the teacher.” In this sample, the Cronbach’s alpha coefficients for this construct were 0.63 at the pre-test and 0.67 at the post-test.

#### Self-compassion scale (short form) for children.

To assess students’ self-compassion, we utilized the Self-Compassion Scale for Children [57] (SCS-C). The SCS-C was developed for use with children and adolescents by adapting the SCS-Short Form [57], which primarily has been used with adults. Items from the SCS-SF were modified to use with younger populations by altering the language to be age-appropriate. For example, Item 1 on the SCS-SF, “When I fail at something important to me, I become consumed by feelings of inadequacy,” was changed to “When I fail at something important to me, I feel like I’m not good enough” [58]. The measure consists of 12 items assessing self-compassion across six 2-item subscales: self-kindness (e.g., “I try to be kind towards those parts of myself I don’t like”), self-judgment (e.g., “I am hard on myself about my own flaws”), common humanity (e.g., “When I fail at something, I try to remember that everybody fails sometimes too”), isolation (e.g., “When I fail at something that’s important to me, I feel like I’m all alone”), mindfulness (e.g., “When something upsets me I try to stay calm”), and over-identification (e.g., “When I’m feeling down, I can’t stop thinking about everything that’s wrong”). Responses were given on a 5-point Likert-type scale, ranging from 1 (*almost never)* to 5 (*almost always*). Previous work identified two subscales based on factor analysis. Confirmatory factor analysis was used to validate the two-factor structure in this sample. The fit of the two-factor model for self-compassion was found to be good, as indicated by the following fit indices: RMSEA = 0.048, CFI = 0.988, TLI = 0.891, and SRMR = 0.040. The two factors were identified as positive domains of self-compassion (common humanity, mindfulness, and self-kindness) and negative domains of the self-compassion measure (isolation, self-judgment, and overidentification). In this sample, the scale’s internal consistency is reflected in Cronbach’s alphas of 0.83 at the pre-test and 0.81 at the post-test for positive self-compassion. The scale’s internal consistency was also demonstrated via Cronbach’s alphas of 0.82 at the pre-test and 0.81 at the post-test for negative self-compassion.

#### The classroom supportiveness scale.

To assess students’ perceptions of general supportiveness in the classroom, students were asked to respond to the 14-item Classroom Supportiveness subscale of the Sense of Classroom as a Community Scale [59]. This subscale assesses the degree to which students evaluate their classmates as supportive and helpful (e.g., “Students in this class help each other learn”). Students responded to the items using a 5-point Likert-type scale from 1 (*disagree a lot*) to 5 (*agree a lot*). Evidence for the validity and reliability of this subscale has been demonstrated in previous research [59]. For the present study, internal consistency, as assessed via Cronbach alphas, was 0.86 at the pre-test and 0.87 at the post-test.

#### Academic goals questionnaire.

To assess academic growth mindset, students completed the Academic Goals Questionnaire, a measure that assesses students’ academic goals [60]. The measure comprises five items, and students rated each item on a 5-point Likert scale. Example questions include “If I have enough time, I can do a good job on all my schoolwork” and “Even if the work in school is hard, I can learn it.” In this sample, the Cronbach alphas coefficients for this construct were 0.83 at the pre-test and 0.88 at the post-test.

#### Satisfaction with life scale for children.

Students’ life satisfaction was assessed with the 5-item Satisfaction with Life Scale for Children [50] (SWLS-C). Example questions include “In most ways my life is close to the way I would want it to be’ and “If I could live my life over, I would have it the same way.” Students rated the items on a five-point Likert-type scale ranging from 1 (*Disagree a lot*) to 5 (*Agree a lot*). Ratings were averaged to produce a total score with higher scores indicating higher levels of life satisfaction. Evidence supporting the validity and reliability of the SWLS-C has been documented with samples of children and early adolescents [61]. In the present study, Cronbach’s alpha coefficient for this contrast was 0.83 at the pre-test and 0.87 at the post-test.

### Data analysis

#### Missing data.

In our analysis, we handled missing data using multiple imputation. We had less than 10% missing data, accounting for 30 missing at post-test in the intervention group and 11%, or 39, missing at endline in the comparison group. We assumed that the data was missing at random. Based on this level of missing data and the results of this analysis, we determined that 15 imputations would be adequate for the baseline data. To ensure the robustness of our findings, we chose to perform 15 cycles of imputation. This decision was based on using as many imputations as the percentage of incomplete cases, and by the desire to capture the variability introduced by the missing data comprehensively. The imputation method we used was the imputation of means into the dataset, a technique that is well-suited for handling missing at random (MAR) data.

#### Measuring group differences.

In this paper, we conducted a comprehensive analysis to assess the effectiveness of the SEE Learning program, in which 4th and 5th-grade students who received the program were compared to students in a waitlist control group. We conducted an Analysis of Covariance (ANCOVA). For the ANCOVA, we reported the F-statistic and its corresponding p-value to gauge the statistical significance, along with the post hoc estimate of Cohen’s d to quantify the effect size. This provided a robust method to assess the impact of the SEE Learning program, with adjustments for key demographic variables and baseline differences.

#### Baseline equivalence.

In the study, controlling for baseline differences in pre-test responses were achieved using ANCOVA. ANCOVA is a statistical technique that adjusts for initial differences between groups. In this context, it allowed for the comparison of post-test scores between the intervention and control groups while accounting for any baseline discrepancies. By using pre-test scores as a covariate, the ANCOVA analysis effectively controlled for initial group differences on the final outcomes, focusing on the effects of the intervention. The covariate “grade” was included to account for differences observed between the groups at baseline.

## Results

The aims of the present study were twofold: 1) to assess the implementation (fidelity, dosage, and acceptability) of the SEE Learning program, and 2) to examine the preliminary student outcomes of the SEE Learning program on multiple social and emotional competencies.

### Aim 1: Intervention dosage, feasibility, and acceptability

In the implementing classrooms, teachers engaged in the program with minimal preparation time, as all lessons required “30 minutes or less” of preparation, except for Lesson 12, which required “15 minutes or less.” Data were not collected for Learning Experience 11 because the research team was unable to distribute the forms before the implementation week. All teachers (100%) completed at least some of each learning experience, ensuring comprehensive coverage of the curriculum across all implementing classrooms. At a minimum, teachers were expected to implement the check-in and core activity, and this requirement was met in all cases. However, there was variation in the implementation of the reflective practices and debrief components. Lesson fidelity was high, with adherence to lesson plans ranging from 63% to 100%, reflecting a strong commitment to the program standards. Teacher acceptability of the program, as measured by their reported comfort levels, was notably high. Over 85% of lessons received positive ratings from teachers, indicating that they felt well-prepared and confident in delivering the content. Student engagement mirrored this positive trend, with most lessons achieving over 85%, showcasing a highly interactive and engaging learning atmosphere. The average lesson duration spanned 30–50 minutes, with an absolute range of 20–120 minutes. However, most lessons were completed within a 60-minute window of time. These numbers highlight the program’s feasibility in late elementary school (Table 3).

**Table 3:** Implementation feasibility based on weekly teacher feedback from implementing classrooms.

Lesson	Preparation Time^@^	% Complete^$^	Lesson Fidelity (% Moderate High to High)^^^	Teacher Comfort (% Moderate High to High Comfort)^&^	Student Engagement (% Positively Engaged)^&^	Lesson Duration Avg (Min/Max)
**1**	30 min or less	100	85	77	100	41 min (20/60)
**2**	30 min or less	100	85	100	92	40 min (20/60)
**3**	30 min or less	100	92	85	100	45 min (20/60)
**4**	30 min or less	85	91	73	100	30 min (20/60)
**5**	30 min or less	92	91	67	100	50 min (20/120)
**6**	30 min or less	100	85	92	100	30 min (20/60)
**7**	30 min or less	75	100	78	100	40 min (20/60)
**8**	30 min or less	91	82	91	100	50 min (20/60)
**9**	30 min or less	100	63	75	88	42 min (20/60)
**10**	30 min or less	88	86	100	100	35 min (20/60)
**12**	15 min or less	100	80	100	100	30 min (20/30)

@“Preparation Time” indicates the average time teachers spent preparing for the lesson.

$“% Classes Completed” shows the percentage of classes that completed the lesson.

^“Lesson Fidelity” reflects the adherence to the lesson plan, with “Moderate” indicating some deviations.

&“Teacher Comfort” and “Student Engagement” percentages represent the proportion of classes reporting moderately high to high levels of comfort and engagement, respectively.

*“Lesson Duration” provides the average time spent on the lesson, with a range indicating the shortest and longest durations observed.

### Aim 2: Student outcomes

The results of the study, using ANCOVA, demonstrate significant impacts of the SEE Learning program on various domains (Table 4). The intrapersonal domains of perspective- taking, empathic concern, intrinsic prosocial motivation, and self-compassion all demonstrated notable improvements in the intervention group compared to the wait-list control group, as indicated by significant F-statistics and Cohen’s D values. Students’ perception of classroom support decreased in both groups; however, the decline was significantly smaller in the intervention group compared to the control group. The more distal outcome, students’ ability to set academic goals and their perception of achieving them, increased in the intervention group, whereas no such improvement was observed in the waitlist control group. Effect sizes for the intervention ranged from 0.26 to 0.33, indicating small to moderate positive effects consistent with those typically reported in the SEL literature.

**Table 4:** Intervention ANCOVA results table for program impact over time, controlling for grade.

	Intervention (*n *= 314)		Wait-List Control (*n *= 302)		Group Difference		
	Pre-test	Post-test	Pre-test	Post-test	F-Stat	df	Cohen’s D
**Intrapersonal Domains of Impact**							
**Awareness & Appraisal**							
Perspective-taking	2.96 (2.87- 3.04)	3.11 (3.02- 3.19)	3.07 (3.0- 3.15)	2.91 (2.82-2.99)	6.45***	1	0.26
**Emotional Resonance**							
Empathic Concern	3.85 (3.77-3.93)	3.94 (3.77-3.93)	3.93 (3.85-4.00)	3.78 (3.70-3.86)	6.26***	1	0.25
**Tolerating Uncomfortable Feelings**							
Emotional Expression	3.05 (2.94-3.16)	3.14 (3.02-3.25)	3.09 (2.98-3.20)	3.11 (3.01-3.23)	0.40	1	0.06
**Motivation to Act**							
Intrinsic Prosocial Motivation	4.07 (3.98-4.16)	4.14 (4.05-4.23)	4.06 (3.97-4.15)	3.92 (3.82-4.01)	12.11***	1	0.33
**Action**							
Positive Self-Compassion	3.02 (2.93-3.10)	3.03 (2.94-3.12)	3.03 (2.94-3.12)	2.89 (2.80-2.98)	5.15***	1	0.22
Negative Orientation to Self	3.14 (3.03-3.24)	3.12 (3.01-3.22)	3.20 (3.09-3.30)	3.24 (3.13-3.35)	0.48	1	0.07
**Interpersonal Domains of Impact**							
Relationship							
Student Class Supportive	3.51 (3.42-3.60)	3.49 (3.38-3.57)	3.25 (3.16-3.34)	3.16 (3.07-3.25)	10.98***	1	0.34
Student Group Respect	3.83 (3.72-3.93)	3.70 (3.59-3.81)	3.44 (3.33-3.54)	3.26 (3.15-3.36)	2.83	0.43	0.17
**Distal Outcomes**							
Academic Goal Setting	3.63 (3.53-3.74)	3.77 (3.66-3.88)	3.56 (3.46-3.67)	3.55 (3.45-3.66)	8.65***	1	0.28
Life Satisfaction	3.95 (3.84-4.05)	3.98 (3.88-4.09)	3.76 (3.66-3.86)	3.81 (3.70-3.91)	3.96	1	0.19

Standard Deviation in parentheses

***p < .01, **p < .05

## Discussion

This is the first quasi-experimental trial to assess the feasibility and preliminary effects of the SEE Learning program with elementary school students in the United States. Overall, our findings suggest that the SEE Learning program (1) is relatively feasible for teachers to implement and is well-received by both teachers and students; and (2) shows potential to enhance students’ compassion-related responses and academic goal setting. This pilot study supports a model of SEL in which compassion-based practices are associated with positive changes in students’ interpersonal and intrapersonal competencies. A strength of this study is that it evaluated a compassion-based SEL program implemented under “real-world” conditions in diverse classrooms typical of many large school districts. This approach provides ecological validity, as it reflects the actual contexts in which teachers address the needs of a diverse student population in regular classrooms. Assessing the effectiveness of SEL programs within actual classroom settings provides preliminary evidence for the program’s external validity, demonstrating its impact across different contexts.

### Implementation quality

Results regarding the implementation of the program suggested that a relatively easy-to-use and cost-effective SEL program focused on compassion-based practices can be implemented with high fidelity. Responding to the recent call for researchers to include implementation data in evaluations of SEL programs, we specifically designed our study to collect data on teachers’ perceptions of the feasibility and acceptability of the SEE Learning program, as well as their views on student engagement.

Our analysis of the SEE Learning teacher diaries showed that lesson dosage and fidelity were relatively high. All SEE Learning teachers (100%) completed at least some part of each learning experience, with adherence to lesson plans ranging from 63% to 100%. This implementation fidelity was higher than what is typically reported in other evaluations of classroom-based SEL programs. As noted by Durlak and DuPre, “Expecting perfect or near-perfect implementation is unrealistic. Positive results have often been obtained with levels around 60%; few studies have attained levels greater than 80%” [50].

A key takeaway regarding implementation is that the SEE Learning program offers a cost-effective and accessible approach that can be seamlessly integrated into classroom routines, making it a viable resource for schools. The program demonstrated high levels of fidelity and acceptability, suggesting that it aligns well with teachers’ instructional styles and is comfortable to deliver. Notably, even without perfect implementation, the observed adherence to core components and positive outcomes indicates that meaningful benefits are achievable.

The high level of implementation fidelity may be attributed to the supports provided to the SEE Learning program teachers. The curriculum was manualized, and each teacher received an intensive 3-day training led by a certified facilitator. This training included an overview of the program’s framework, well-being practices, experiential training, and planning for classroom implementation. Over 95% of teachers rated the training highly, and all intervention-group teachers attended at least 90% of the sessions.

The implementation fidelity of the SEE Learning curriculum may also be attributed to its implementation during the COVID-19 pandemic, a time when mental health concerns among students were heightened. Given the heightened concern for student well-being, teachers may have been especially committed to implementing the program to address and alleviate students’ elevated stress levels. Future research should explore the implementation fidelity of the SEE Learning program under current conditions and in diverse contexts, including countries outside the U.S.

In addition to the ease of use of the program, teachers reported high levels of student involvement in the SEE Learning lessons; for 9 out of the 12 lessons, 100% of students were reported to be positively engaged. Although teachers’ perceptions of student engagement may not be as informative as the students’ own views, these insights provide valuable information about teachers’ experiences with their students’ engagement and interest in the program.

### Impacts

The findings of this study indicate modest but statistically significant positive effects of the SEE Learning program on several social and emotional competencies among elementary school students. Students in the intervention group demonstrated significant improvements in perspective-taking, empathic concern, and self-compassion, whereas students in the waitlist control group showed declines in these areas. This divergence suggests that SEE Learning exerted both promotive and protective effects, bolstering competencies while also buffering against normative declines observed during middle childhood [54].

Effect sizes for these gains ranged from 0.26 to 0.33, reflecting small to moderate effects consistent with meta-analyses of school-based SEL programs [10]. Beyond core empathy-related skills, students exposed to the SEE Learning curriculum also reported increased intrinsic prosocial motivation, greater perceptions of classroom supportiveness, enhanced self-compassion, and stronger academic goal-setting. These outcomes align with broader SEL scholarship, which underscores the value of nurturing emotional awareness, interpersonal competencies, and classroom climate to support holistic student development [4,13].

The observed gains in intrinsic prosocial motivation resonate with self-determination theory, which posits that when educational contexts foster autonomy, competence, and relatedness, students are more likely to internalize prosocial values and behaviors [62]. Likewise, improvements in self-compassion and goal-setting illustrate how SEL interventions can support both emotional resilience and academic engagement. Taken together, these results suggest that SEE Learning meaningfully strengthens key intrapersonal and interpersonal skills, contributing to students’ overall social-emotional development in a context-sensitive and evidence-aligned manner.

These findings also suggest that the SEE Learning program may play a pivotal role in counteracting the normative decline in empathy-related responses that have been observed in elementary-aged students. This pattern, documented in prior developmental research [21,63], highlights the importance of timely interventions that sustain and strengthen prosocial tendencies. It also may suggest that this type of intervention or the skills fostered within this type of intervention might be a critical component that, when absent, allows a schooling experience to negatively impact student prosocial development. Our results provide empirical support for SEE Learning as a protective intervention that not only halts this downward trajectory but enhances children’s empathic concern, perspective-taking, and intrinsic prosocial motivation.

While these outcomes parallel findings from other established SEL interventions, such as Roots of Empathy (ROE), which have demonstrated significant gains in helping, sharing, cooperation, and emotional understanding [21], this study extends the literature by focusing on a program with an explicit compassion-centered framework. Unlike many SEL models that emphasize self-awareness and emotion regulation as primary levers, SEE Learning integrates compassion as both a guiding principle and instructional objective. Our findings suggest that cultivating compassion in early education may yield unique benefits for children’s internal prosocial drive, beyond externally motivated behaviors.

By demonstrating increases in students’ internalized desire to help others, this study contributes to a growing body of research exploring the motivational mechanisms underlying prosocial behavior. In doing so, it helps fill a gap in the SEL literature, which has historically focused more on behavioral manifestations of prosociality than on students’ autonomous motivations. This supports recent calls in the field for greater attention to the quality and origins of student motivation in SEL outcomes [66].

The observed gains may stem from SEE Learning’s pedagogical design, which weaves compassion-related discourse, mindfulness-based emotional regulation, and collaborative classroom practices into its core lessons. This holistic approach appears to foster both intrapersonal reflection and interpersonal understanding, consistent with emerging frameworks that view SEL not only as a skill-building endeavor but as a relational and ethical practice [7].

Notably, the mixed findings on classroom supportiveness introduce important questions for future inquiry. Although students in SEE Learning classrooms reported relatively higher perceptions of supportiveness post-intervention, the overall decline across both groups complicates interpretation. These results may reflect measurement limitations, contextual disruptions such as COVID-19-related peer interaction constraints, or unmeasured classroom dynamics. Incorporating observational data or constructs more directly aligned with SEE Learning’s theory of change could enhance construct validity and interpretation.

Finally, the observed increase in academic growth mindset contributes to expanding dialogue on the academic spillover effects of SEL. While prior work has established links between empathy, classroom climate, and academic outcomes [10,13], the current findings add nuance by highlighting how compassion-based SEL approaches may bolster students’ motivational beliefs. In doing so, SEE Learning positions itself as a dual-impact intervention, supporting both emotional well-being and academic engagement through compassion-driven pedagogy.

## Limitations

Several limitations are important to note about this study. First, the use of a quasi-experimental design rather than a randomized controlled trial constrains our ability to assess causal program impacts. The study was conducted during the post-COVID return to school, and school administrators were hesitant to offer the program to only a portion of their students. Consequently, while schools agreed to participate in either the intervention or wait-listed control groups, they did not permit randomization at the classroom level. This recruitment method may have biased both groups towards schools that were highly motivated to implement the program, making it difficult to generalize the findings to schools with moderate or low motivation. This major limitation can be mitigated, in part, by the fact that the patterns of change observed across several dimensions of social behavior align with the criteria outlined by Shadish, Cook, and Campbell [61] for making causal claims in quasi-experimental designs. Notably, our significant findings regarding perspective-taking, empathic concern, self-compassion, intrinsic prosocial motivation, and academic goal setting showed patterns of change from pre-test to post-test in opposite directions for the SEE Learning and wait-list control groups, suggesting that alternative explanations for these results are unlikely.

Another limitation of our study is that analyses were conducted at the individual child level, even though the selection was made at the classroom level. Unfortunately, the small number of classrooms did not provide enough statistical power for multi-level modeling. This clustering of children within classrooms creates non-independence among subjects, which could bias the statistical tests used to assess intervention effects. This is a common challenge in evaluating universal, school-based interventions when resources are insufficient to recruit a large number of classrooms or schools [61]. Nonetheless, as noted by Slavin, although analyzing data at the child level when randomization is done at the classroom level is discouraged by methodologists because the findings can exaggerate statistical significance “... their effect sizes are unbiased [64] and therefore are of value... ” (p. 9) [65].

## Conclusion and directions for future research

This study demonstrated the preliminary impacts of the SEE Learning program in the Southeastern region of the USA. Results showed positive effects across various measures of social and emotional competencies, including perspective-taking, empathic concern, self-compassion, and intrinsic prosocial motivation. These results highlight the potential of compassion training to foster intrapersonal growth and promote positive social and emotional development among elementary school students. Future research should delve deeper into the reasons behind the observed trends, exploring potential mediating and moderating factors. Randomized control studies with longitudinal follow-up are needed to assess the long-term effects of the SEE Learning intervention on students’ social and emotional development, well-being, and academic achievement to determine whether the positive impacts are durable.

## Supporting information

S1 Table Teacher feedback diaries. This is the table of feedback that each teacher was asked to complete after the delivery of a learning experience. This data was used to determine fidelity, acceptability, and engagement.
(DOCX)

S2 Table Student measures key. This table is a list of all measures that were administered to all student participants.
(DOCX)
