# Supplementary material for: Evaluating the feasibility and preliminary impact of the Social, Emotional, and Ethical (SEE) Learning program: A compassion-based social and emotional learning program for elementary school children
Source: PLoS One. 2025 Aug 13;20(8):e0328519. doi: 10.1371/journal.pone.0328519 (PMC12349709; doi:10.1371/journal.pone.0328519)
Supplement: S1 Table — This is the table of feedback that each teacher was asked to complete after the delivery of a learning experience. This data was used to determine fidelity, acceptability, and engagement. (DOCX) [file pone.0328519.s001.docx]

**SEE Learning Program**

**Implementation Feedback Diary**

**Teachers**

**SEE LEARNING TEACHER NAME____­­­­­­­­___________________________**

**SCHOOL____________________________________________________**

**GRADE_____________________________________________________**

| Week 1 - Chapter 1 - Learning Experience 1 - Exploring Kindness | | | |
| --- | --- | --- | --- |
| Date Delivery Started ________________________ | | **Date Delivery Completed______________________** | |
| Lesson Components  (Please check those completed)   - Check in - Presentation/Discussion - Insight Activity - Reflective Practice - Debrief | **About how much time did you spend preparing for the lesson?**   - None - < 15 min - 15-30 mins - 30-60 mins - 1-2 hours | | **About how much time did you spend implementing the lesson?**   - < 30 mins - 30-60 mins - 1-2 hours - 3-4 hours |
| How closely did you follow the lesson as described in the lesson plan? Please provide a rating:   - VERY HIGH: Followed the activity as prescribed (80%-100%). - MODERATELY HIGH: Followed most of the activity directions (60%-80%) - AVERAGE: Followed about half (40%-60%) of the activity directions. - MODERATELY LOW: Followed less than half (about 20%-40%) of the activity directions. - VERY LOW: Did not follow most of the activity directions (less than 20%).   If you did not follow the lessons very highly then why? And did you do something else? If so What? | | | |
| How comfortable did you feel while facilitating this learning experience? | | | |
| - VERY COMFORTABLE - MODERATELY HIGH COMFORT - AVERAGE COMFORT - MODERATELY LOW COMFORT - VERY LOW COMFORT | | | |
| Level of Student Engagement   - 1 – not engaged - 2 - 3 – somewhat engaged - 4 - 5 – very highly engaged   Do you think your students had this level of engagement?: | | | |
| Did you notice any positive or negative response in your students? (you may select both but please reflect on this)   Yes  No   Positive or  Negative  Comments: | | | |
| Would you implement this learning experience again?   Yes   No   Maybe  Comments: | | | |
| Please discuss how teaching the lesson felt compared to how you normally deliver lessons. Similarities? Differences? | | | |
| What differences, if any, did you notice in the way you felt with your class after the lesson? | | | |

| Week 2 - Chapter 1 - Learning Experience 2 – CLASS AGREEMENTS | | | |
| --- | --- | --- | --- |
| Date Delivery Started ________________________ | | **Date Delivery Completed______________________** | |
| Lesson Components  (Please check those completed)   - Check in - Presentation/Discussion - Insight Activity - Reflective Practice - Debrief | **About how much time did you spend preparing for the lesson?**   - None - < 15 min - 15-30 mins - 30-60 mins - 1-2 hours | | **About how much time did you spend implementing the lesson?**   - < 30 mins - 30-60 mins - 1-2 hours - 3-4 hours |
| How closely did you follow the lesson as described in the lesson plan? Please provide a rating:   - VERY HIGH: Followed the activity as prescribed (80%-100%). - MODERATELY HIGH: Followed most of the activity directions (60%-80%) - AVERAGE: Followed about half (40%-60%) of the activity directions. - MODERATELY LOW: Followed less than half (about 20%-40%) of the activity directions. - VERY LOW: Did not follow most of the activity directions (less than 20%).   If you did not follow the lessons very highly then why? And did you do something else? If so What? | | | |
| How comfortable did you feel while facilitating this learning experience? | | | |
| - VERY COMFORTABLE - MODERATELY HIGH COMFORT - AVERAGE COMFORT - MODERATELY LOW COMFORT - VERY LOW COMFORT | | | |
| Level of Student Engagement   - 1 – not engaged - 2 - 3 – somewhat engaged - 4 - 5 – very highly engaged   Do you think your students had this level of engagement?: | | | |
| Did you notice any positive or negative response in your students? (you may select both but please reflect on this)   Yes  No   Positive or  Negative  Comments: | | | |
| Would you implement this learning experience again?   Yes   No   Maybe  Comments: | | | |
| Please discuss how teaching the lesson felt compared to how you normally deliver lessons. Similarities? Differences? | | | |
| What differences, if any, did you notice in the way you felt with your class after the lesson? | | | |

| Week 3 - Chapter 1 - Learning Experience 3– Practicing kindness | | | |
| --- | --- | --- | --- |
| Date Delivery Started ________________________ | | **Date Delivery Completed______________________** | |
| Lesson Components  (Please check those completed)   - Check in - Presentation/Discussion - Insight Activity - Reflective Practice - Debrief | **About how much time did you spend preparing for the lesson?**   - None - < 15 min - 15-30 mins - 30-60 mins - 1-2 hours | | **About how much time did you spend implementing the lesson?**   - < 30 mins - 30-60 mins - 1-2 hours - 3-4 hours |
| How closely did you follow the lesson as described in the lesson plan? Please provide a rating:   - VERY HIGH: Followed the activity as prescribed (80%-100%). - MODERATELY HIGH: Followed most of the activity directions (60%-80%) - AVERAGE: Followed about half (40%-60%) of the activity directions. - MODERATELY LOW: Followed less than half (about 20%-40%) of the activity directions. - VERY LOW: Did not follow most of the activity directions (less than 20%).   If you did not follow the lessons very highly then why? And did you do something else? If so What? | | | |
| How comfortable did you feel while facilitating this learning experience? | | | |
| - VERY COMFORTABLE - MODERATELY HIGH COMFORT - AVERAGE COMFORT - MODERATELY LOW COMFORT - VERY LOW COMFORT | | | |
| Level of Student Engagement   - 1 – not engaged - 2 - 3 – somewhat engaged - 4 - 5 – very highly engaged   Do you think your students had this level of engagement?: | | | |
| Did you notice any positive or negative response in your students? (you may select both but please reflect on this)   Yes  No   Positive or  Negative  Comments: | | | |
| Would you implement this learning experience again?   Yes   No   Maybe  Comments: | | | |
| Please discuss how teaching the lesson felt compared to how you normally deliver lessons. Similarities? Differences? | | | |
| What differences, if any, did you notice in the way you felt with your class after the lesson? | | | |

| Week 4 - Chapter 1 - Learning Experience 4 – Kindness as an inner quality | | | |
| --- | --- | --- | --- |
| Date Delivery Started ________________________ | | **Date Delivery Completed______________________** | |
| Lesson Components  (Please check those completed)   - Check in - Presentation/Discussion - Insight Activity - Reflective Practice - Debrief | **About how much time did you spend preparing for the lesson?**   - None - < 15 min - 15-30 mins - 30-60 mins - 1-2 hours | | **About how much time did you spend implementing the lesson?**   - < 30 mins - 30-60 mins - 1-2 hours - 3-4 hours |
| How closely did you follow the lesson as described in the lesson plan? Please provide a rating:   - VERY HIGH: Followed the activity as prescribed (80%-100%). - MODERATELY HIGH: Followed most of the activity directions (60%-80%) - AVERAGE: Followed about half (40%-60%) of the activity directions. - MODERATELY LOW: Followed less than half (about 20%-40%) of the activity directions. - VERY LOW: Did not follow most of the activity directions (less than 20%).   If you did not follow the lessons very highly then why? And did you do something else? If so please explain. | | | |
| How comfortable did you feel while facilitating this learning experience? | | | |
| - VERY COMFORTABLE - MODERATELY HIGH COMFORT - AVERAGE COMFORT - MODERATELY LOW COMFORT - VERY LOW COMFORT | | | |
| Level of Student Engagement   - 1 – not engaged - 2 - 3 – somewhat engaged - 4 - 5 – very highly engaged   Do you think your students had this level of engagement?: | | | |
| Did you notice any positive or negative response in your students? (you may select both but please reflect on this)   Yes  No   Positive or  Negative  Comments: | | | |
| Would you implement this learning experience again?   Yes   No   Maybe  Comments: | | | |
| Please discuss how teaching the lesson felt compared to how you normally deliver lessons. Similarities? Differences? | | | |
| What differences, if any, did you notice in the way you felt with your class after the lesson? | | | |

| Week 5 - Chapter 1 - Learning Experience 5 – recognizing kindness & exploring INTERDEPENDENCE | | | |
| --- | --- | --- | --- |
| Date Delivery Started ________________________ | | **Date Delivery Completed______________________** | |
| Lesson Components  (Please check those completed)   - Check in - Presentation/Discussion - Insight Activity - Reflective Practice - Debrief | **About how much time did you spend preparing for the lesson?**   - None - < 15 min - 15-30 mins - 30-60 mins - 1-2 hours | | **About how much time did you spend implementing the lesson?**   - < 30 mins - 30-60 mins - 1-2 hours - 3-4 hours |
| How closely did you follow the lesson as described in the lesson plan? Please provide a rating:   - VERY HIGH: Followed the activity as prescribed (80%-100%). - MODERATELY HIGH: Followed most of the activity directions (60%-80%) - AVERAGE: Followed about half (40%-60%) of the activity directions. - MODERATELY LOW: Followed less than half (about 20%-40%) of the activity directions. - VERY LOW: Did not follow most of the activity directions (less than 20%).   If you did not follow the lessons very highly then why? And did you do something else? If so What? | | | |
| How comfortable did you feel while facilitating this learning experience? | | | |
| - VERY COMFORTABLE - MODERATELY HIGH COMFORT - AVERAGE COMFORT - MODERATELY LOW COMFORT - VERY LOW COMFORT | | | |
| Level of Student Engagement   - 1 – not engaged - 2 - 3 – somewhat engaged - 4 - 5 – very highly engaged   Do you think your students had this level of engagement?: | | | |
| Did you notice any positive or negative response in your students? (you may select both but please reflect on this)   Yes  No   Positive or  Negative  Comments: | | | |
| Would you implement this learning experience again?   Yes   No   Maybe  Comments: | | | |
| Please discuss how teaching the lesson felt compared to how you normally deliver lessons. Similarities? Differences? | | | |
| What differences, if any, did you notice in the way you felt with your class after the lesson? | | | |

| Week 6 - Chapter 2 - Learning Experience 1 – exploring sensations | | | |
| --- | --- | --- | --- |
| Date Delivery Started ________________________ | | **Date Delivery Completed______________________** | |
| Lesson Components  (Please check those completed)   - Check in - Presentation/Discussion - Insight Activity - Reflective Practice - Debrief | **About how much time did you spend preparing for the lesson?**   - None - < 15 min - 15-30 mins - 30-60 mins - 1-2 hours | | **About how much time did you spend implementing the lesson?**   - < 30 mins - 30-60 mins - 1-2 hours - 3-4 hours |
| How closely did you follow the lesson as described in the lesson plan? Please provide a rating:   - VERY HIGH: Followed the activity as prescribed (80%-100%). - MODERATELY HIGH: Followed most of the activity directions (60%-80%) - AVERAGE: Followed about half (40%-60%) of the activity directions. - MODERATELY LOW: Followed less than half (about 20%-40%) of the activity directions. - VERY LOW: Did not follow most of the activity directions (less than 20%).   If you did not follow the lessons very highly then why? And did you do something else? If so What? | | | |
| How comfortable did you feel while facilitating this learning experience? | | | |
| - VERY COMFORTABLE - MODERATELY HIGH COMFORT - AVERAGE COMFORT - MODERATELY LOW COMFORT - VERY LOW COMFORT | | | |
| Level of Student Engagement   - 1 – not engaged - 2 - 3 – somewhat engaged - 4 - 5 – very highly engaged   Do you think your students had this level of engagement?: | | | |
| Did you notice any positive or negative response in your students? (you may select both but please reflect on this)   Yes  No   Positive or  Negative  Comments: | | | |
| Would you implement this learning experience again?   Yes   No   Maybe  Comments: | | | |
| Please discuss how teaching the lesson felt compared to how you normally deliver lessons. Similarities? Differences? | | | |
| What differences, if any, did you notice in the way you felt with your class after the lesson? | | | |

| Week 7 - Chapter 2 - Learning Experience 2 – Resourcing | | | |
| --- | --- | --- | --- |
| Date Delivery Started ________________________ | | **Date Delivery Completed______________________** | |
| Lesson Components  (Please check those completed)   - Check in - Presentation/Discussion - Insight Activity - Reflective Practice - Debrief | **About how much time did you spend preparing for the lesson?**   - None - < 15 min - 15-30 mins - 30-60 mins - 1-2 hours | | **About how much time did you spend implementing the lesson?**   - < 30 mins - 30-60 mins - 1-2 hours - 3-4 hours |
| How closely did you follow the lesson as described in the lesson plan? Please provide a rating:   - VERY HIGH: Followed the activity as prescribed (80%-100%). - MODERATELY HIGH: Followed most of the activity directions (60%-80%) - AVERAGE: Followed about half (40%-60%) of the activity directions. - MODERATELY LOW: Followed less than half (about 20%-40%) of the activity directions. - VERY LOW: Did not follow most of the activity directions (less than 20%).   If you did not follow the lessons very highly then why? And did you do something else? If so What? | | | |
| How comfortable did you feel while facilitating this learning experience? | | | |
| - VERY COMFORTABLE - MODERATELY HIGH COMFORT - AVERAGE COMFORT - MODERATELY LOW COMFORT - VERY LOW COMFORT | | | |
| Level of Student Engagement   - 1 – not engaged - 2 - 3 – somewhat engaged - 4 - 5 – very highly engaged   Do you think your students had this level of engagement?: | | | |
| Did you notice any positive or negative response in your students? (you may select both but please reflect on this)   Yes  No   Positive or  Negative  Comments: | | | |
| Would you implement this learning experience again?   Yes   No   Maybe  Comments: | | | |
| Please discuss how teaching the lesson felt compared to how you normally deliver lessons. Similarities? Differences? | | | |
| What differences, if any, did you notice in the way you felt with your class after the lesson? | | | |

| Week 8 - Chapter 2 - Learning Experience 3 – Creating a Treasure chest | | | |
| --- | --- | --- | --- |
| Date Delivery Started ________________________ | | **Date Delivery Completed______________________** | |
| Lesson Components  (Please check those completed)   - Check in - Presentation/Discussion - Insight Activity - Reflective Practice - Debrief | **About how much time did you spend preparing for the lesson?**   - None - < 15 min - 15-30 mins - 30-60 mins - 1-2 hours | | **About how much time did you spend implementing the lesson?**   - < 30 mins - 30-60 mins - 1-2 hours - 3-4 hours |
| How closely did you follow the lesson as described in the lesson plan? Please provide a rating:   - VERY HIGH: Followed the activity as prescribed (80%-100%). - MODERATELY HIGH: Followed most of the activity directions (60%-80%) - AVERAGE: Followed about half (40%-60%) of the activity directions. - MODERATELY LOW: Followed less than half (about 20%-40%) of the activity directions. - VERY LOW: Did not follow most of the activity directions (less than 20%).   If you did not follow the lessons very highly then why? And did you do something else? If so What? | | | |
| How comfortable did you feel while facilitating this learning experience? | | | |
| - VERY COMFORTABLE - MODERATELY HIGH COMFORT - AVERAGE COMFORT - MODERATELY LOW COMFORT - VERY LOW COMFORT | | | |
| Level of Student Engagement   - 1 – not engaged - 2 - 3 – somewhat engaged - 4 - 5 – very highly engaged   Do you think your students had this level of engagement?: | | | |
| Did you notice any positive or negative response in your students? (you may select both but please reflect on this)   Yes  No   Positive or  Negative  Comments: | | | |
| Would you implement this learning experience again?   Yes   No   Maybe  Comments: | | | |
| Please discuss how teaching the lesson felt compared to how you normally deliver lessons. Similarities? Differences? | | | |
| What differences, if any, did you notice in the way you felt with your class after the lesson? | | | |

| Week 9 - Chapter 2 - Learning Experience 4 - Grounding | | | |
| --- | --- | --- | --- |
| Date Delivery Started ________________________ | | **Date Delivery Completed______________________** | |
| Lesson Components  (Please check those completed)   - Check in - Presentation/Discussion - Insight Activity - Reflective Practice - Debrief | **About how much time did you spend preparing for the lesson?**   - None - < 15 min - 15-30 mins - 30-60 mins - 1-2 hours | | **About how much time did you spend implementing the lesson?**   - < 30 mins - 30-60 mins - 1-2 hours - 3-4 hours |
| How closely did you follow the lesson as described in the lesson plan? Please provide a rating:   - VERY HIGH: Followed the activity as prescribed (80%-100%). - MODERATELY HIGH: Followed most of the activity directions (60%-80%) - AVERAGE: Followed about half (40%-60%) of the activity directions. - MODERATELY LOW: Followed less than half (about 20%-40%) of the activity directions. - VERY LOW: Did not follow most of the activity directions (less than 20%).   If you did not follow the lessons very highly then why? And did you do something else? If so What? | | | |
| How comfortable did you feel while facilitating this learning experience? | | | |
| - VERY COMFORTABLE - MODERATELY HIGH COMFORT - AVERAGE COMFORT - MODERATELY LOW COMFORT - VERY LOW COMFORT | | | |
| Level of Student Engagement   - 1 – not engaged - 2 - 3 – somewhat engaged - 4 - 5 – very highly engaged   Do you think your students had this level of engagement?: | | | |
| Did you notice any positive or negative response in your students? (you may select both but please reflect on this)   Yes  No   Positive or  Negative  Comments: | | | |
| Would you implement this learning experience again?   Yes   No   Maybe  Comments: | | | |
| Please discuss how teaching the lesson felt compared to how you normally deliver lessons. Similarities? Differences? | | | |
| What differences, if any, did you notice in the way you felt with your class after the lesson? | | | |

| Week 10 - Chapter 2 - Learning Experience 5 – Resilience zone | | | |
| --- | --- | --- | --- |
| Date Delivery Started ________________________ | | **Date Delivery Completed______________________** | |
| Lesson Components  (Please check those completed)   - Check in - Presentation/Discussion - Insight Activity - Reflective Practice - Debrief | **About how much time did you spend preparing for the lesson?**   - None - < 15 min - 15-30 mins - 30-60 mins - 1-2 hours | | **About how much time did you spend implementing the lesson?**   - < 30 mins - 30-60 mins - 1-2 hours - 3-4 hours |
| How closely did you follow the lesson as described in the lesson plan? Please provide a rating:   - VERY HIGH: Followed the activity as prescribed (80%-100%). - MODERATELY HIGH: Followed most of the activity directions (60%-80%) - AVERAGE: Followed about half (40%-60%) of the activity directions. - MODERATELY LOW: Followed less than half (about 20%-40%) of the activity directions. - VERY LOW: Did not follow most of the activity directions (less than 20%).   If you did not follow the lessons very highly then why? And did you do something else? If so What? | | | |
| How comfortable did you feel while facilitating this learning experience? | | | |
| - VERY COMFORTABLE - MODERATELY HIGH COMFORT - AVERAGE COMFORT - MODERATELY LOW COMFORT - VERY LOW COMFORT | | | |
| Level of Student Engagement   - 1 – not engaged - 2 - 3 – somewhat engaged - 4 - 5 – very highly engaged   Do you think your students had this level of engagement?: | | | |
| Did you notice any positive or negative response in your students? (you may select both but please reflect on this)   Yes  No   Positive or  Negative  Comments: | | | |
| Would you implement this learning experience again?   Yes   No   Maybe  Comments: | | | |
| Please discuss how teaching the lesson felt compared to how you normally deliver lessons. Similarities? Differences? | | | |
| What differences, if any, did you notice in the way you felt with your class after the lesson? | | | |

| Week 11 - Chapter 2 - Learning Experience 6 – resilience zone continued | | | |
| --- | --- | --- | --- |
| Date Delivery Started ________________________ | | **Date Delivery Completed______________________** | |
| Lesson Components  (Please check those completed)   - Check in - Presentation/Discussion - Insight Activity - Reflective Practice - Debrief | **About how much time did you spend preparing for the lesson?**   - None - < 15 min - 15-30 mins - 30-60 mins - 1-2 hours | | **About how much time did you spend implementing the lesson?**   - < 30 mins - 30-60 mins - 1-2 hours - 3-4 hours |
| How closely did you follow the lesson as described in the lesson plan? Please provide a rating:   - VERY HIGH: Followed the activity as prescribed (80%-100%). - MODERATELY HIGH: Followed most of the activity directions (60%-80%) - AVERAGE: Followed about half (40%-60%) of the activity directions. - MODERATELY LOW: Followed less than half (about 20%-40%) of the activity directions. - VERY LOW: Did not follow most of the activity directions (less than 20%).   If you did not follow the lessons very highly then why? And did you do something else? If so What? | | | |
| How comfortable did you feel while facilitating this learning experience? | | | |
| - VERY COMFORTABLE - MODERATELY HIGH COMFORT - AVERAGE COMFORT - MODERATELY LOW COMFORT - VERY LOW COMFORT | | | |
| Level of Student Engagement   - 1 – not engaged - 2 - 3 – somewhat engaged - 4 - 5 – very highly engaged   Do you think your students had this level of engagement?: | | | |
| Did you notice any positive or negative response in your students? (you may select both but please reflect on this)   Yes  No   Positive or  Negative  Comments: | | | |
| Would you implement this learning experience again?   Yes   No   Maybe  Comments: | | | |
| Please discuss how teaching the lesson felt compared to how you normally deliver lessons. Similarities? Differences? | | | |
| What differences, if any, did you notice in the way you felt with your class after the lesson? | | | |

| Week 12 - Chapter 2 - Learning Experience 7 – How compassion & safety effect the body | | | |
| --- | --- | --- | --- |
| Date Delivery Started ________________________ | | **Date Delivery Completed______________________** | |
| Lesson Components  (Please check those completed)   - Check in - Presentation/Discussion - Insight Activity - Reflective Practice - Debrief | **About how much time did you spend preparing for the lesson?**   - None - < 15 min - 15-30 mins - 30-60 mins - 1-2 hours | | **About how much time did you spend implementing the lesson?**   - < 30 mins - 30-60 mins - 1-2 hours - 3-4 hours |
| How closely did you follow the lesson as described in the lesson plan? Please provide a rating:   - VERY HIGH: Followed the activity as prescribed (80%-100%). - MODERATELY HIGH: Followed most of the activity directions (60%-80%) - AVERAGE: Followed about half (40%-60%) of the activity directions. - MODERATELY LOW: Followed less than half (about 20%-40%) of the activity directions. - VERY LOW: Did not follow most of the activity directions (less than 20%).   If you did not follow the lessons very highly then why? And did you do something else? If so What? | | | |
| How comfortable did you feel while facilitating this learning experience? | | | |
| - VERY COMFORTABLE - MODERATELY HIGH COMFORT - AVERAGE COMFORT - MODERATELY LOW COMFORT - VERY LOW COMFORT | | | |
| Level of Student Engagement   - 1 – not engaged - 2 - 3 – somewhat engaged - 4 - 5 – very highly engaged   Do you think your students had this level of engagement?: | | | |
| Did you notice any positive or negative response in your students? (you may select both but please reflect on this)   Yes  No   Positive or  Negative  Comments: | | | |
| Would you implement this learning experience again?   Yes   No   Maybe  Comments: | | | |
| Please discuss how teaching the lesson felt compared to how you normally deliver lessons. Similarities? Differences? | | | |
| What differences, if any, did you notice in the way you felt with your class after the lesson? | | | |

| Week 13 - Chapter 3 - Learning Experience 1 – exploring the mind | | | |
| --- | --- | --- | --- |
| Date Delivery Started ________________________ | | **Date Delivery Completed______________________** | |
| Lesson Components  (Please check those completed)   - Check in - Presentation/Discussion - Insight Activity - Reflective Practice - Debrief | **About how much time did you spend preparing for the lesson?**   - None - < 15 min - 15-30 mins - 30-60 mins - 1-2 hours | | **About how much time did you spend implementing the lesson?**   - < 30 mins - 30-60 mins - 1-2 hours - 3-4 hours |
| How closely did you follow the lesson as described in the lesson plan? Please provide a rating:   - VERY HIGH: Followed the activity as prescribed (80%-100%). - MODERATELY HIGH: Followed most of the activity directions (60%-80%) - AVERAGE: Followed about half (40%-60%) of the activity directions. - MODERATELY LOW: Followed less than half (about 20%-40%) of the activity directions. - VERY LOW: Did not follow most of the activity directions (less than 20%).   If you did not follow the lessons very highly then why? And did you do something else? If so What? | | | |
| How comfortable did you feel while facilitating this learning experience? | | | |
| - VERY COMFORTABLE - MODERATELY HIGH COMFORT - AVERAGE COMFORT - MODERATELY LOW COMFORT - VERY LOW COMFORT | | | |
| Level of Student Engagement   - 1 – not engaged - 2 - 3 – somewhat engaged - 4 - 5 – very highly engaged   Do you think your students had this level of engagement?: | | | |
| Did you notice any positive or negative response in your students? (you may select both but please reflect on this)   Yes  No   Positive or  Negative  Comments: | | | |
| Would you implement this learning experience again?   Yes   No   Maybe  Comments: | | | |
| Please discuss how teaching the lesson felt compared to how you normally deliver lessons. Similarities? Differences? | | | |
| What differences, if any, did you notice in the way you felt with your class after the lesson? | | | |

| Week 14 - Chapter 3 - Learning Experience 2 – exploring attention | | | |
| --- | --- | --- | --- |
| Date Delivery Started ________________________ | | **Date Delivery Completed______________________** | |
| Lesson Components  (Please check those completed)   - Check in - Presentation/Discussion - Insight Activity - Reflective Practice - Debrief | **About how much time did you spend preparing for the lesson?**   - None - < 15 min - 15-30 mins - 30-60 mins - 1-2 hours | | **About how much time did you spend implementing the lesson?**   - < 30 mins - 30-60 mins - 1-2 hours - 3-4 hours |
| How closely did you follow the lesson as described in the lesson plan? Please provide a rating:   - VERY HIGH: Followed the activity as prescribed (80%-100%). - MODERATELY HIGH: Followed most of the activity directions (60%-80%) - AVERAGE: Followed about half (40%-60%) of the activity directions. - MODERATELY LOW: Followed less than half (about 20%-40%) of the activity directions. - VERY LOW: Did not follow most of the activity directions (less than 20%).   If you did not follow the lessons very highly then why? And did you do something else? If so What? | | | |
| How comfortable did you feel while facilitating this learning experience? | | | |
| - VERY COMFORTABLE - MODERATELY HIGH COMFORT - AVERAGE COMFORT - MODERATELY LOW COMFORT - VERY LOW COMFORT | | | |
| Level of Student Engagement   - 1 – not engaged - 2 - 3 – somewhat engaged - 4 - 5 – very highly engaged   Do you think your students had this level of engagement?: | | | |
| Did you notice any positive or negative response in your students? (you may select both but please reflect on this)   Yes  No   Positive or  Negative  Comments: | | | |
| Would you implement this learning experience again?   Yes   No   Maybe  Comments: | | | |
| Please discuss how teaching the lesson felt compared to how you normally deliver lessons. Similarities? Differences? | | | |
| What differences, if any, did you notice in the way you felt with your class after the lesson? | | | |

| Week 15 - Chapter 3 - Learning Experience 3 – Cultivating attention in activities: part 1 | | | |
| --- | --- | --- | --- |
| Date Delivery Started ________________________ | | **Date Delivery Completed______________________** | |
| Lesson Components  (Please check those completed)   - Check in - Presentation/Discussion - Insight Activity - Reflective Practice - Debrief | **About how much time did you spend preparing for the lesson?**   - None - < 15 min - 15-30 mins - 30-60 mins - 1-2 hours | | **About how much time did you spend implementing the lesson?**   - < 30 mins - 30-60 mins - 1-2 hours - 3-4 hours |
| How closely did you follow the lesson as described in the lesson plan? Please provide a rating:   - VERY HIGH: Followed the activity as prescribed (80%-100%). - MODERATELY HIGH: Followed most of the activity directions (60%-80%) - AVERAGE: Followed about half (40%-60%) of the activity directions. - MODERATELY LOW: Followed less than half (about 20%-40%) of the activity directions. - VERY LOW: Did not follow most of the activity directions (less than 20%).   If you did not follow the lessons very highly then why? And did you do something else? If so What? | | | |
| How comfortable did you feel while facilitating this learning experience? | | | |
| - VERY COMFORTABLE - MODERATELY HIGH COMFORT - AVERAGE COMFORT - MODERATELY LOW COMFORT - VERY LOW COMFORT | | | |
| Level of Student Engagement   - 1 – not engaged - 2 - 3 – somewhat engaged - 4 - 5 – very highly engaged   Do you think your students had this level of engagement?: | | | |
| Did you notice any positive or negative response in your students? (you may select both but please reflect on this)   Yes  No   Positive or  Negative  Comments: | | | |
| Would you implement this learning experience again?   Yes   No   Maybe  Comments: | | | |
| Please discuss how teaching the lesson felt compared to how you normally deliver lessons. Similarities? Differences? | | | |
| What differences, if any, did you notice in the way you felt with your class after the lesson? | | | |

| Week 16 - Chapter 3 - Learning Experience 4 – Cultivating attention in activities: part 2 | | | |
| --- | --- | --- | --- |
| Date Delivery Started ________________________ | | **Date Delivery Completed______________________** | |
| Lesson Components  (Please check those completed)   - Check in - Presentation/Discussion - Insight Activity - Reflective Practice - Debrief | **About how much time did you spend preparing for the lesson?**   - None - < 15 min - 15-30 mins - 30-60 mins - 1-2 hours | | **About how much time did you spend implementing the lesson?**   - < 30 mins - 30-60 mins - 1-2 hours - 3-4 hours |
| How closely did you follow the lesson as described in the lesson plan? Please provide a rating:   - VERY HIGH: Followed the activity as prescribed (80%-100%). - MODERATELY HIGH: Followed most of the activity directions (60%-80%) - AVERAGE: Followed about half (40%-60%) of the activity directions. - MODERATELY LOW: Followed less than half (about 20%-40%) of the activity directions. - VERY LOW: Did not follow most of the activity directions (less than 20%).   If you did not follow the lessons very highly then why? And did you do something else? If so What? | | | |
| How comfortable did you feel while facilitating this learning experience? | | | |
| - VERY COMFORTABLE - MODERATELY HIGH COMFORT - AVERAGE COMFORT - MODERATELY LOW COMFORT - VERY LOW COMFORT | | | |
| Level of Student Engagement   - 1 – not engaged - 2 - 3 – somewhat engaged - 4 - 5 – very highly engaged   Do you think your students had this level of engagement?: | | | |
| Did you notice any positive or negative response in your students? (you may select both but please reflect on this)   Yes  No   Positive or  Negative  Comments: | | | |
| Would you implement this learning experience again?   Yes   No   Maybe  Comments: | | | |
| Please discuss how teaching the lesson felt compared to how you normally deliver lessons. Similarities? Differences? | | | |
| What differences, if any, did you notice in the way you felt with your class after the lesson? | | | |

| Week 17 - Chapter 3 - Learning Experience 5 – Cultivating attention on an object | | | |
| --- | --- | --- | --- |
| Date Delivery Started ________________________ | | **Date Delivery Completed______________________** | |
| Lesson Components  (Please check those completed)   - Check in - Presentation/Discussion - Insight Activity - Reflective Practice - Debrief | **About how much time did you spend preparing for the lesson?**   - None - < 15 min - 15-30 mins - 30-60 mins - 1-2 hours | | **About how much time did you spend implementing the lesson?**   - < 30 mins - 30-60 mins - 1-2 hours - 3-4 hours |
| How closely did you follow the lesson as described in the lesson plan? Please provide a rating:   - VERY HIGH: Followed the activity as prescribed (80%-100%). - MODERATELY HIGH: Followed most of the activity directions (60%-80%) - AVERAGE: Followed about half (40%-60%) of the activity directions. - MODERATELY LOW: Followed less than half (about 20%-40%) of the activity directions. - VERY LOW: Did not follow most of the activity directions (less than 20%).   If you did not follow the lessons very highly then why? And did you do something else? If so What? | | | |
| How comfortable did you feel while facilitating this learning experience? | | | |
| - VERY COMFORTABLE - MODERATELY HIGH COMFORT - AVERAGE COMFORT - MODERATELY LOW COMFORT - VERY LOW COMFORT | | | |
| Level of Student Engagement   - 1 – not engaged - 2 - 3 – somewhat engaged - 4 - 5 – very highly engaged   Do you think your students had this level of engagement?: | | | |
| Did you notice any positive or negative response in your students? (you may select both but please reflect on this)   Yes  No   Positive or  Negative  Comments: | | | |
| Would you implement this learning experience again?   Yes   No   Maybe  Comments: | | | |
| Please discuss how teaching the lesson felt compared to how you normally deliver lessons. Similarities? Differences? | | | |
| What differences, if any, did you notice in the way you felt with your class after the lesson? | | | |

| Week 18 - Chapter 3 - Learning Experience 6 – self-awareness | | | |
| --- | --- | --- | --- |
| Date Delivery Started ________________________ | | **Date Delivery Completed______________________** | |
| Lesson Components  (Please check those completed)   - Check in - Presentation/Discussion - Insight Activity - Reflective Practice - Debrief | **About how much time did you spend preparing for the lesson?**   - None - < 15 min - 15-30 mins - 30-60 mins - 1-2 hours | | **About how much time did you spend implementing the lesson?**   - < 30 mins - 30-60 mins - 1-2 hours - 3-4 hours |
| How closely did you follow the lesson as described in the lesson plan? Please provide a rating:   - VERY HIGH: Followed the activity as prescribed (80%-100%). - MODERATELY HIGH: Followed most of the activity directions (60%-80%) - AVERAGE: Followed about half (40%-60%) of the activity directions. - MODERATELY LOW: Followed less than half (about 20%-40%) of the activity directions. - VERY LOW: Did not follow most of the activity directions (less than 20%).   If you did not follow the lessons very highly then why? And did you do something else? If so What? | | | |
| How comfortable did you feel while facilitating this learning experience? | | | |
| - VERY COMFORTABLE - MODERATELY HIGH COMFORT - AVERAGE COMFORT - MODERATELY LOW COMFORT - VERY LOW COMFORT | | | |
| Level of Student Engagement   - 1 – not engaged - 2 - 3 – somewhat engaged - 4 - 5 – very highly engaged   Do you think your students had this level of engagement?: | | | |
| Did you notice any positive or negative response in your students? (you may select both but please reflect on this)   Yes  No   Positive or  Negative  Comments: | | | |
| Would you implement this learning experience again?   Yes   No   Maybe  Comments: | | | |
| Please discuss how teaching the lesson felt compared to how you normally deliver lessons. Similarities? Differences? | | | |
| What differences, if any, did you notice in the way you felt with your class after the lesson? | | | |

| Week 19 – additional Learning EXPERIENCES | | | |
| --- | --- | --- | --- |
| Name of Learning Experience _________________ | | **Date Delivery Started ________________________**  **Date Delivery Completed______________________** | |
| Lesson Components  (Please check those completed)   - Check in - Presentation/Discussion - Insight Activity - Reflective Practice - Debrief | **About how much time did you spend preparing for the lesson?**   - None - < 15 min - 15-30 mins - 30-60 mins - 1-2 hours | | **About how much time did you spend implementing the lesson?**   - < 30 mins - 30-60 mins - 1-2 hours - 3-4 hours |
| How closely did you follow the lesson as described in the lesson plan? Please provide a rating:   - VERY HIGH: Followed the activity as prescribed (80%-100%). - MODERATELY HIGH: Followed most of the activity directions (60%-80%) - AVERAGE: Followed about half (40%-60%) of the activity directions. - MODERATELY LOW: Followed less than half (about 20%-40%) of the activity directions. - VERY LOW: Did not follow most of the activity directions (less than 20%).   If you did not follow the lessons very highly then why? And did you do something else? If so What? | | | |
| How comfortable did you feel while facilitating this learning experience? | | | |
| - VERY COMFORTABLE - MODERATELY HIGH COMFORT - AVERAGE COMFORT - MODERATELY LOW COMFORT - VERY LOW COMFORT | | | |
| Level of Student Engagement   - 1 – not engaged - 2 - 3 – somewhat engaged - 4 - 5 – very highly engaged   Do you think your students had this level of engagement?: | | | |
| Did you notice any positive or negative response in your students? (you may select both but please reflect on this)   Yes  No   Positive or  Negative  Comments: | | | |
| Would you implement this learning experience again?   Yes   No   Maybe  Comments: | | | |
| Please discuss how teaching the lesson felt compared to how you normally deliver lessons. Similarities? Differences? | | | |
| What differences, if any, did you notice in the way you felt with your class after the lesson? | | | |

| Week 20 – additional Learning EXPERIENCES | | | |
| --- | --- | --- | --- |
| Name of Learning Experience _________________ | | **Date Delivery Started ________________________**  **Date Delivery Completed______________________** | |
| Lesson Components  (Please check those completed)   - Check in - Presentation/Discussion - Insight Activity - Reflective Practice - Debrief | **About how much time did you spend preparing for the lesson?**   - None - < 15 min - 15-30 mins - 30-60 mins - 1-2 hours | | **About how much time did you spend implementing the lesson?**   - < 30 mins - 30-60 mins - 1-2 hours - 3-4 hours |
| How closely did you follow the lesson as described in the lesson plan? Please provide a rating:   - VERY HIGH: Followed the activity as prescribed (80%-100%). - MODERATELY HIGH: Followed most of the activity directions (60%-80%) - AVERAGE: Followed about half (40%-60%) of the activity directions. - MODERATELY LOW: Followed less than half (about 20%-40%) of the activity directions. - VERY LOW: Did not follow most of the activity directions (less than 20%).   If you did not follow the lessons very highly then why? And did you do something else? If so What? | | | |
| How comfortable did you feel while facilitating this learning experience? | | | |
| - VERY COMFORTABLE - MODERATELY HIGH COMFORT - AVERAGE COMFORT - MODERATELY LOW COMFORT - VERY LOW COMFORT | | | |
| Level of Student Engagement   - 1 – not engaged - 2 - 3 – somewhat engaged - 4 - 5 – very highly engaged   Do you think your students had this level of engagement?: | | | |
| Did you notice any positive or negative response in your students? (you may select both but please reflect on this)   Yes  No   Positive or  Negative  Comments: | | | |
| Would you implement this learning experience again?   Yes   No   Maybe  Comments: | | | |
| Please discuss how teaching the lesson felt compared to how you normally deliver lessons. Similarities? Differences? | | | |
| What differences, if any, did you notice in the way you felt with your class after the lesson? | | | |
