# Supplementary material for: Evaluating the feasibility and preliminary impact of the Social, Emotional, and Ethical (SEE) Learning program: A compassion-based social and emotional learning program for elementary school children
Source: PLoS One. 2025 Aug 13;20(8):e0328519. doi: 10.1371/journal.pone.0328519 (PMC12349709; doi:10.1371/journal.pone.0328519)
Supplement: S2 Table — This table is a list of all measures that were administered to all student participants. (DOCX) [file pone.0328519.s002.docx]

| **Student**  **MEASURES KEY**  (Items in order that they appear in survey) | | | |
| --- | --- | --- | --- |
| **ITEMS AS THEY APPEAR ON SURVEY** | **RESPONSE FORMAT** | **NOTES ON ITEMS**  Sub-scales; reverse scored items | **ORIGINAL SURVEY INFO**  References, original item numbers, and response formats from original survey |
| **PART 1 – WHITE SURVEY** | | | |
| **Demographics**  What **grade** are you in this year?  How do you describe your **gender**?  What is your **birthdate**?  **How old are you?**  Family composition  **Which adults do you live with most of the time**? (examples: mother and father, part time with each parent, foster parent or caregiver, two mothers, father and father’s girlfriend or boyfriend, grandmother)  **How many brothers or sisters do you have? ________**  **What is the first language you learned at home?** *(*You can check more than one if you need to.)  **Which language(s) do you speak at home?** *(*You can check more than one if you need to.)  **Which language do you prefer to speak?** *(*You can check more than one if you need to.)  English proficiency  **How difficult is it for you to read in English?** | (Circle one)  4  5  6  7  (Circle one)  Boy  Girl  In another way  The way I describe my gender is ______.  (Write in)  Month / Day / Year  ______(write in)  □ Mother  □ Father  □ Stepfather  □ Stepmother  □ Part time with each parent  □ Foster parent(s) or caregiver(s)  □ Other adults (write in the space, for example, grandparents, second mom or dad, mom’s boyfriend or girlfriend, dad’s boyfriend or girlfriend): ___________________  Fill in the blank  □ English □ Korean  □ Cantonese □ Mandarin  □ Spanish  □ Hindi  □ Other, please specify:___________________  □ Very hard  □ Hard  □ Easy  □ Very easy |  | Note: the order of Grade and Gender questions are switched since the response format for Gender is different for this survey from previous surveys.  Taken from MDI instructional survey |
| **Sample questions:**  I like to eat pizza.  I like to eat carrots. |  |  |  |

| **ITEMS AS THEY APPEAR ON SURVEY** | **RESPONSE FORMAT** | **NOTES ON ITEMS**  Sub-scales; reverse scored items | **ORIGINAL SURVEY INFO**  References, original item numbers, and response formats from original survey |
| --- | --- | --- | --- |
| **EMPATHY/COMPASSION MEASURE = My Feelings About Others** | | | |
| **Interpersonal Reactivity Index- 14 Items**  **(Empathic Concern and Perspective-taking subscales)**   1. I often feel sorry for people who don’t have the things I have. 2. It’s easy for me to understand why other people do the things they do. 3. Sometimes I feel very sorry for other people when they are having problems. 4. When I see someone being picked on, I feel kind of sorry for them. 5. Sometimes I try to understand my friends better by imagining how they think about things. 6. Even when I’m mad at someone, I try to understand how they feel. 7. I often feel sorry for other children who are sad or in trouble. 8. I try to understand how other kids feel before I decide what to say to them. 9. When I see someone being treated mean, it bothers me. 10. Even when I know I’m right I listen to what other people think. 11. I often have strong feelings about things that happen around me. 12. Before I say anything bad about anyone, I try to imagine how I would feel if I were that person. 13. I am a person who cares about the feelings of others. 14. There are different ways to think about a problem and I try to look at all of them. | *Response format modified from original.*  Please indicate how much these statements describe you::  1 Not at all like me  2 A little bit like me  3 Kind of like me  4 A lot like me  5 Always like me | *All items modified from the original by Miller.*  **Empathy:** 1, 3, 4, 7, 9, 11, 13  **Perspective-taking:** 2, 5, 6, 8, 10, 12, 14  **No reverse scored items.** | Davis, M. H. (1983). Measuring individual differences in empathy: Evidence for a multidimensional approach. *Journal of Personality and Social Psychology, 44*, 113-126. (modified by Miller, personal communication)  **Original Items-**  Empathic Concern: 2, 4*, 9, 14*, 18*, 20, 22,  Perspective Taking: 3*, 8, 11, 15*, 21, 25, 28  * Items Reverse Scored  **Original Response Format**  0 Does not describe me well  4 Describes me very well |

| **ITEMS AS THEY APPEAR ON SURVEY** | **RESPONSE FORMAT** | **NOTES ON ITEMS**  Sub-scales; reverse scored items | **ORIGINAL SURVEY INFO**  References, original item numbers, and response formats from original survey |
| --- | --- | --- | --- |
| **PROSOCIALITY MEASURES= WHAT MAKES YOU WANT TO HELP** | | | |
| **Intrinsic/**  **Extrinsic Prosocial Motivation**  1. Because I think it is good to help**. (1P)**  2. So I will get help in return **(EP).**  3. Because I would feel bad if I didn’t help. **(1P)**  4. Because I want to get a reward or praise from the teacher.**(EP).**  5. Because the teacher told me to help. **(EP).**  6. Because I am concerned about the other person. **(1P)** | **When you help another student in your class, why do you usually do it?**  1 Disagree a lot  2 Disagree a little  3 Don’t agree or disagree  4 Agree a little  5 Agree a lot  **Original response:**  When you help another student in your class, why do you usually do it?  1. Often  2. Hardly Ever  3. Sometimes  4. Often  5. Always | **Intrinsic Prosocial Motivation**: 1, 3, 6  **Extrinsic Prosocial Motivation**: 2, 4, 5 | **Developmental Studies Questionnaire – From Student Scales**  Retrieved from [www.devstu.org](http://www.devstu.org)  [Dev Studies Questonnaires.pdf](file:///C:\Users\Jenna\Dropbox%20(KSR%20Lab)\UBC%20Epigenetics%20SEL%20Study\Measures%20to%20Print%20in%20Lab\RAK%20Measures\Dev%20Studies%20Questonnaires.pdf)  NOTE: Intrinsic Motivation is often scored as the ratio of the intrinsic to the extrinsic  score (or as the proportion of the sum of the intrinsic items to the sum of intrinsic plus the  extrinsic items).  **Omitted Items:**  When you try not to make noise in this class why do you usually do it?  1. Often  2. Hardly Ever  3. Sometimes  4. Often  5. Always   1. Because it would be wrong to disturb other students who are trying to work. **(IP)** 2. Because I would feel bad if my noisiness stopped someone else from learning something. **(IP)** 3. Because I am thinking about how I would feel if I was trying to concentrate, and others were making noise. **(IP)** 4. So I (or my group) will get points or a prize if I (we) can keep quiet. **(EP)** 5. Because I’ll get in trouble if I make noise. **(EP)** 6. Because the teacher told us to be quiet.**(EP)** |

| **ITEMS AS THEY APPEAR ON SURVEY** | **RESPONSE FORMAT** | **NOTES ON ITEMS**  Sub-scales; reverse scored items | **ORIGINAL SURVEY INFO**  References, original item numbers, and response formats from original survey |
| --- | --- | --- | --- |
| **RESILIENCY MEASURES= My Many Feelings** | | | |
| **Emotional Expression Scale for Children (EESC) - kept 6 items**   1. I have feelings that I can’t figure out 2. When I feel upset, I do not know how to talk about it 3. I often do not know how I am feeling 4. People tell me I should talk about my feelings more often 5. Sometimes I just don’t have words to describe how I feel 6. I often do not know why I am angry | **How true is each statement for you?**  **1 not at all true**  **2 a little true**  **3 somewhat true**  **4 very true**  **5 extremely true** | **Poor awareness factor**  **Removing some items (#1, 7 – i.e., items < .6 FA)** | Penza-Clyve, S., & Zeman, J. (2002). Initial validation of the emotion expression scale for children (EESC). *Journal of Clinical Child and Adolescent Psychology*, *31*(4), 540-547.  Validated with 4-5^th^ grade students  ORIGINAL   1. When something bad happens, I feel like exploding 2. I have feelings that I can’t figure out 3. When I feel upset, I do not know how to talk about it 4. I often do not know how I am feeling 5. People tell me I should talk about my feelings more often 6. Sometimes I just don’t have words to describe how I feel 7. I know I should show my feelings, but it’s too hard 8. I often do not know why I am angry |

| **ITEMS AS THEY APPEAR ON SURVEY** | **RESPONSE FORMAT** | **NOTES ON ITEMS**  Sub-scales; reverse scored items | **ORIGINAL SURVEY INFO**  References, original item numbers, and response formats from original survey |
| --- | --- | --- | --- |
| **SELF-COMPASSION MEASURE= HOW I FEEL ABOUT MYSELF** | | | |
| **Self-compassion Scale (short form) - 12 items**   1. When I fail at something important to me, I feel like I’m not good enough. (R) 2. I try to be kind towards those things about myself I don’t like. 3. When something bad happens, I try not to focus only on the bad, but think about the good things as well. 4. When I’m feeling sad, I feel like most other kids are happier than I am. (R) 5. When I fail at something, I try to remember that everybody fails sometimes too. 6. When I’m going through a very hard time, I’m really nice to myself. 7. When something upsets me I try to stay calm**.** 8. When I fail at something that’s important to me, I feel like I’m all alone. (R) 9. When I’m feeling sad, I can’t stop thinking about everything that’s wrong. (R) 10. When I feel like I’m not good enough at something, I try to remind myself that everyone feels that  way sometimes. 11. I am hard on myself about my own flaws/weaknesses. (R) 12. I get frustrated or upset about the things about myself I don’t like. (R) | **How often do you do the following?**  1 Never  2 Almost Never  3 Sometimes  4 Almost Always  5 Always | **Self-Kindness**: 2, 6  **Self-Judgment**: 11, 12  **Common Humanity**: 5, 10  **Isolation**: 4, 8  **Mindfulness**: 3, 7  **Over-identified**: 1, 9  **Reverse scored items***:* 1, 4, 8, 9, 11, 12  **No subscales to be used for Short Form (12 items)** | Raes, F., Pommier, E., Neff, K. D., & Van Gucht, D. (2011). Construction and factorial validation of a short form of the Self-Compassion Scale.  *Clinical Psychology & Psychotherapy. 18*, 250-255.  All items modified by Lawlor, 2011.  **Items from Original Measure:**  1. When I fail at something important to me I become consumed by feelings of inadequacy.*  2. I try to be understanding and patient towards those aspects of my personality I don’t like.  3. When something painful happens I try to take a balanced view of the situation.  4. When I’m feeling down, I tend to feel like most other people are probably happier than I am.*  5. I try to see my failings as part of the human condition.  6. When I am going through a very hard time, I give myself the caring and tenderness I need.  7. When something upsets me I try to keep my emotions in balance.  8. When I fail at something that’s important, I tend to feel alone in my failure.*  9. When I’m feeling down, I tend to obsess and fixate on everything that’s wrong.*  10. When I feel adequate in some way, I try to remind myself that feelings of inadequacy are shared by most people.  11. I’m disapproving and judgmental about my own flaws and inadequacies.*  12. I’m intolerant and impatient towards those aspects of my personality I don’t like.*  *Reverse Scored Items |

| **ITEMS AS THEY APPEAR ON SURVEY** | **RESPONSE FORMAT** | **NOTES ON ITEMS**  Sub-scales; reverse scored items | **ORIGINAL SURVEY INFO**  References, original item numbers, and response formats from original survey |  |
| --- | --- | --- | --- | --- |
| **WELL-BEING MEASURE= My Life** | | | |  |
| **Satisfaction with Life Scale for Children –**  **5 items**   1. In most ways my life is close to the way I would want it to be. 2. The things in my life are excellent. 3. I am happy with life. 4. So far I have gotten the important things I want in life. 5. If I could live my life over, I would have it the same way. | **How true is each statement for you?**  1 Disagree a lot  2 Disagree a little  3 Don’t agree or disagree  4 Agree a little  5 Agree a lot | **No reverse scored items** | Gadermann, A. M., Schonert-Reichl, K. A., & Zumbo, B. D. (2010). Investigating validity evidence of the *Satisfaction with Life* Scale adapted for Children. *Social Indicators Research, 96,* 229–247  Items appear exactly as in original measure.  Directions in orginal measure are:  “For each of the following statements, please circle the number that describes you the best.” |  |
| **How happy are you with your life as a whole these days?** | **Please circle one number (either under or in between the faces) Not at all happy – Very Happy 1-10** | *Open ended* |  | IN ORIGINAL |
| 1. In the space below, list things in your life that you are thankful for or make you happy.   List as many things as you can.   1. What are some ways to show kindness to others? List as many as you can think of in the space below. |  | *Open ended* |  |  |
